# Interactive Bioinformatics Lab: Using Genomic Databases for Active Learning in Dentistry

**DOI:** 10.1002/jdd.13911

**Published:** 2025-04-25

**Authors:** François Isnaldo Dias Caldeira, Raquel Mantuaneli Scarel‐Caminaga

**Affiliations:** ^1^ Department of Diagnosis and Surgery São Paulo State University‐UNESP School of Dentistry Araraquara Brazil; ^2^ Department of Morphology Genetics, Orthodontics and Pediatric Dentistry São Paulo State University‐UNESP School of Dentistry Araraquara Brazil

## 1 Problem

With the advance of artificial intelligence and the availability of infinite petabytes of information, bioinformatics has become central to solving important contemporary research challenges [1, 2]. However, graduate and postgraduate dental students did not have many opportunities to be in contact with bioinformatics, given the nature of dentistry, which demands much dedication to clinics. Students who need to focus on basic research to connect it with dentistry have enormous benefits in learning bioinformatics. Opportunities for active learning using bioinformatic tools in the dental field are rare.

## 2 Solution

**FIGURE 1:**
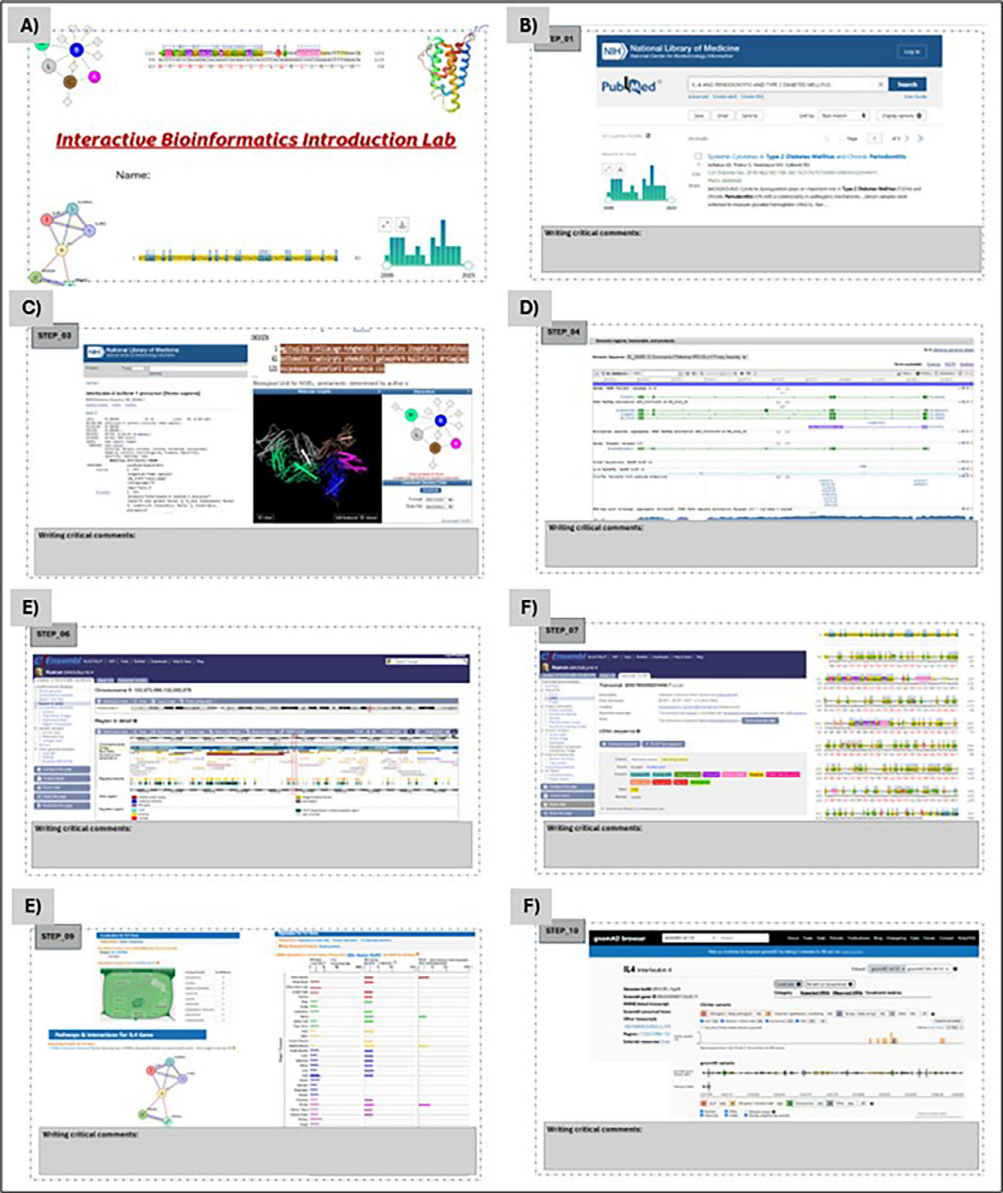
Interactive bioinformatics laboratory model using PowerPoint slides for active learning. Illustrative model so that students can carry out their search. (A–F) Representative screenshots of the stages developed in the interactive lab. All queries for information in the respective databases were made on March 10, 2025. Detailed steps on how to obtain the results can be found in Supplementary Material S1.

We developed the pedagogical activity “Interactive Bioinformatics Introduction Laboratory” to create an active learning resource for graduate and postgraduate dental students, to introduce them to, or improve their knowledge of, bioinformatics and handle genomic databases. This interactive teaching–learning resource consists in:

- Give students a script/guide containing all the information needed to carry out the activity (Table 1) and a practical guide to the steps to be followed (Figure_1), detailed in Supplementary material_1.
- Explain the activity in the classroom while the materials (Table 1 and Supplementary material 1) are presented.
- Encourage the students to visit and explore the genomic databases in their own notebooks and give help to them when they call.
- Give students a file to be used to paste the obtained set of print screens of the found results (Supplementary material 02), answering the questions of each step (and adding their critical comments).
- On the last page, there are two questions to hear feedback on the activity carried out [3].

**TABLE 1:** Guide for the interactive lab on introduction to bioinformatics.

***Instructions***
This interactive bioinformatics lab includes key steps to help you become familiar with the subject. To achieve this, you will need to create an interactive Word document, incorporating screenshots of the databases you access. Choose a Gene of your interest. You can select a gene by researching a disease of your interest, starting with Google Scholar (https://scholar.google.com.br/) or with PubMed = > https://pubmed.ncbi.nlm.nih.gov/. Search for example (in the search bar): Periodontitis AND Genes. In the results you could be interested in, for example: **IL4**. Then, suppose you wish to research the ***IL4* gene** (Interleukin 4, IL‐4 protein). To conduct a more in‐depth study of the selected gene, explore the databases according to the following steps. Utilizing the file (supplementary material 02) answer what is questioned in each step and write critical comments concisely. On the first page “Interactive Bioinformatics Introduction Lab,” complete the required information.
**STEP_01**:
Since you choose the ***IL4*** gene of your interest, search for a paper that focuses on the gene you choose. Utilize the PubMed = > https://pubmed.ncbi.nlm.nih.gov/. In the Search bar type, for example: IL4. You may add other words to refine your search for studies of your interest. For example, include names of diseases, or syndromes, or countries, etc, like: IL4 AND Periodontitis AND Type 2 diabetes mellitusChoose and access one of the articles. In the supplementary material 02, paste the print‐screen of the PubMed showing the chosen article (with the Journal and year published)**Write comments about the more relevant aspects presented in the Abstract of the paper above, and after, your personal critical comments/perceptions about that study (**what you understood and found interesting)Note: Add the page link to the PowerPoint file.
**STEP_02**:
The first activity in the repository will involve familiarizing yourself with the tool. To do this, access the **NCBI** website and click on **“About NCBI” = >** http://www.ncbi.nlm.nih.gov/. You can “navigate” through any link on this page, explore, and have fun. However, for the purpose of recording the activity for this lesson, it is suggested that you open each link in a new tab so you don't lose track of your progress. Go back to NCBI, open it in another tab, and click **= > Gene = > Search**. Next, click on IL4 – Interleukin 4. https://www.ncbi.nlm.nih.gov/gene/3565 Gene ID: 3565, updated on 4‐Jan‐2025 See the information like: **Gene type ‐**protein coding; **RefSeq status**: REVIEWED. **What are these data for your selected gene?** Roll page down, observe the Genomic data Viewer: **Location**: 5q31.1; **Exon count**:5 Roll page down until **Genomic regions, transcripts, and products**. In **Genomic Sequence** select = > **RefSeqGene** (the gene sequence defined as the most accurate in the database). See the graphic representation of the exons and introns, and provide a screenshot showing a segment (e.g., the first 1000 base pairs) of a nucleotide sequence, and include the webpage link. Then, answer the questions. Note: Add the page link to the PowerPoint file.
**STEP_03**:
Notice the tab titled **“Related information”**. Click on **Protein** to access the protein tab, where the corresponding sequence will be displayed. Then, select **Protein 3D Structure** to view the three‐dimensional structure of the protein in question.For proteins referenced in **RefSeq proteins**, such as **IL‐4**, there are three isoforms of this protein.For example, **Interleukin‐4 isoform 1 precursor [Homo sapiens]**, which consists of 153 amino acids, has the following identifiers: **Accession**: NP_000580.1 **GI**: 4504669 Access one of the isoforms of the protein encoded by your gene of interest. After that, capture a screenshot of the relevant information and write a comment about the interesting aspects you observed, following a procedure similar to the one described in step 1.Note: Add the page link to the PowerPoint file.
**STEP_04**:
Return to the **NCBI** homepage and open a new tab. Click on **Gene**, then select **Search**. Next, choose **Genome**. This will display the organisms for which this gene sequence is available in the database. Take a screenshot showing this information. After identifying an organism, click on its **gene** to view detailed sequencing information. Finally, write a comment about the diversity of organisms you observed and their associated gene sequences. Note: Add the page link to the PowerPoint file.
**STEP_05**:
Go to NCBI in Clinical. Check **dbSNP** (it shows the number of SNPs in this gene). Note this in the comments, then enter the page and provide the link to it. Take a screenshot of the first screen. Observe how the information for each SNP is displayed. You can enter one of them to see more details. Go back to **NCBI** and navigate to the Clinical section. Select **OMIM** (Online Mendelian Inheritance in Man) and open it in a new tab. Locate and select 147780 – INTERLEUKIN 4; IL4, then click on the link. Take a screenshot of the page and observe the vast amount of information available about this gene, including links to other pages for further exploration. The items on the **OMIM** page related to this gene are listed in the left‐hand menu. Review these sections and write a comment about the wealth of details and connections provided for this gene. Note: Add the page link to the PowerPoint file.
**STEP_06**:
In **OMIM**, select **Genome: Ensembl** (European Molecular Biology Laboratory) from the options on the right‐hand menu. This will redirect you to another database, which is highly illustrative. Take a screenshot of the page and comment on the information available about this gene, including links to other pages. Focus on aspects such as the gene's chromosomal location, its proximity to other genes, the gene sequences, mRNA, protein sequences, and their associated functions. Note: Add the page link to the PowerPoint file.
**STEP_07**:
Return to **OMIM** and select the **DNA**: **Ensembl** link. This will redirect you to the DNA section of the Ensembl database. Take a screenshot of the page and comment on the aligned and color‐coded sequence of the gene, as well as the sequences displayed below for mRNA and protein. Pay special attention to polymorphic sites and mutation types, which are highlighted in different colors. Note: Add the page link to the PowerPoint file.
**STEP_08**:
Return to **OMIM** and select the **Protein: UniProt link**. This will direct you to a detailed page containing numerous links with information about the protein, including its isoforms and functions. Take a screenshot of the page, specifically showing the diagram of the **subcellular localization** of the protein. Write a comment discussing the protein, its subcellular localization, and any relevant features or functions highlighted in the **UniProt** database. Note: Add the page link to the PowerPoint file.
**STEP_09**:
Return to **OMIM** and select the **Gene Info link**. From there, choose **GeneCards**—an excellent resource that compiles all available information about the gene through links to various related pages.Take a screenshot of the section you find most interesting and write a comment discussing your observations.Note: GeneCards includes links to companies specializing in biomolecular products related to this gene.In the Gene Info section, there are additional useful sites (screenshots not required): **BioGPS**: Provides details on where the gene is expressed, the protein's functions, and includes Wikipedia‐derived information. **Ensembl**: Offers data on transcripts and proteins, as well as visualizations of the gene's structure (exons/introns). **NCBI**: Features gene mapping, sequences, expression data, structure, function, references, citations, and homology. **KEGG** (Kyoto Encyclopedia of Genes and Genomes): Contains signaling pathway diagrams, protein structure and functions, and related data. For example, it links the drug Suplatast Tosylate, a Th2 cytokine inhibitor (targeting IL‐4 [HSA:3565] and IL‐5 [HSA:3567]) to asthma mechanisms (PATH:hsa05310). Note: Add the page link to the PowerPoint file.
**STEP_10**:
Return to **OMIM** and select the **Variation link**. This will open a list of sites, including: **1000 Genomes**: Stores large‐scale sequencing data, primarily polymorphisms. **ClinVar**: Focuses on clinical variants. **gnomAD**: Contains exome data. **GWAS Central**: Specializes in genome‐wide association studies. **HGVS**: Offers standardized variant nomenclature. **NHLBI EVS**: Provides exome variant data from the NHLBI's Exome Sequencing Project. **PharmGKB**: A database dedicated to pharmacogenomics, particularly useful for understanding genetic influences on drug responses. Choose one of these databases, take a screenshot of the page, and write a comment explaining why you selected it. Highlight its specific features and relevance to your research or interests.Note: Add the page link to the PowerPoint file.
**FINAL_QUESTIONS**:
**1**. How this activity has impacted your learning and how can you use it in your studies? **2**. There were difficulties to execute this activity?

## 3 Results

Table 1 of the Interactive Bioinformatics Lab includes detailed explanations of each step, with links and examples, allowing students to develop self‐directed learning in bioinformatics. Figure 1 illustrates what is explained in Table 1, and alternatively, the students may consult Supplementary material 01 to see in detail the practical guide to be followed.

The register of the web pages consulted in each step is facilitated by using the interactive construction of the PowerPoint file (Supplementary material 02), which contains all the steps, answers, and critical comments. This allows the students to problematize the knowledge gathered during the subject of fundamentals of genetics applied to graduate and postgraduate.

**FIGURE 2:**
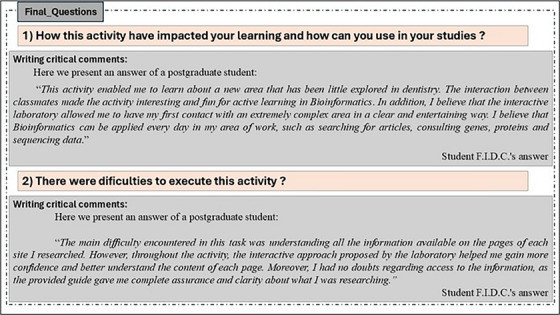
Feedback about how the students felt the impact of the activity on their learning.

The answers obtained from the last page of Supplementary material 01, at the end of the laboratory, provided us important feedback about how the students felt the impact of the activity on their learning and professional impact. Moreover, to know whether the guidance of the use of these tools and navigation by the websites worked out, it was made the final second question (Figure 2).

This active learning approach is effective since students construct their own critical knowledge [3, 4]. We did not find another bioinformatics learning activity like the one presented here, and since there is a critical need for bioinformatics expertise in the life sciences [5], we hope to encourage other educators to utilize this interactive tool in the classroom to teach necessary bioinformatics skills and competencies.

We conclude that this active learning can help graduate students understand bioinformatics and, even more, use its tools to enrich their knowledge and diversify applications.

## Supporting information



Supporting Information

Supporting Information
