# Supplementary material for: Interactive Bioinformatics Lab: Using Genomic Databases for Active Learning in Dentistry
Source: J Dent Educ. 2025 Apr 25;89(Suppl 3):1854–8. doi: 10.1002/jdd.13911 (PMC12728793; doi:10.1002/jdd.13911)
Supplement: Supplementary file 1 — Supporting Information [file JDD-89-1854-s001.pdf]

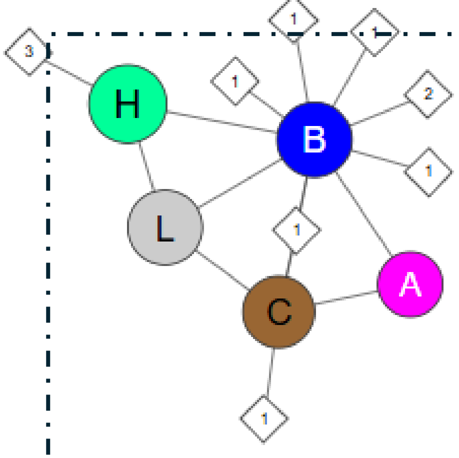

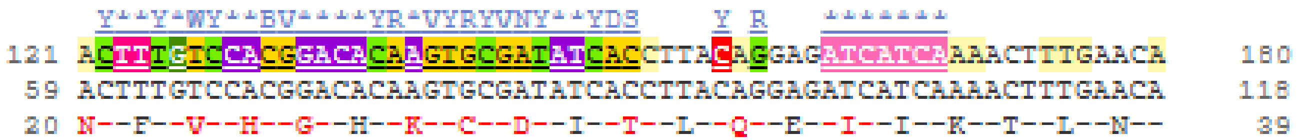

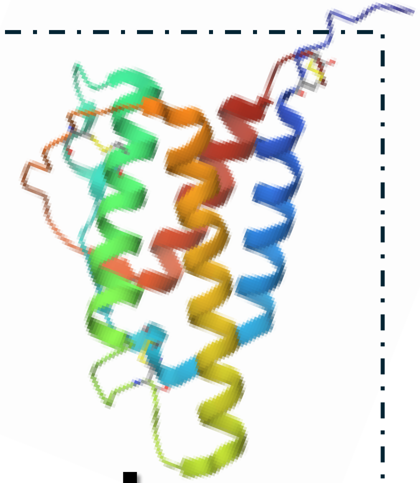

Practical guide to the steps to be followed:

Interactive Bioinformatics Introduction Lab

Name: F.I.D.C .

Chosen Gene: Interleukin -4 (*IL4*) .

Reason(s) for this choice: I am interested on the complexity of imune system, and I have seeing this molecule in papers related with inflammatory diseases.

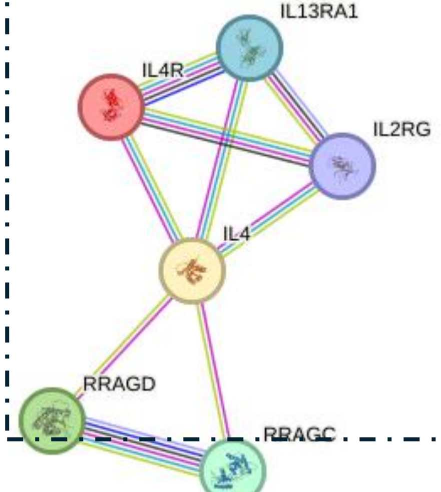

STEP_01

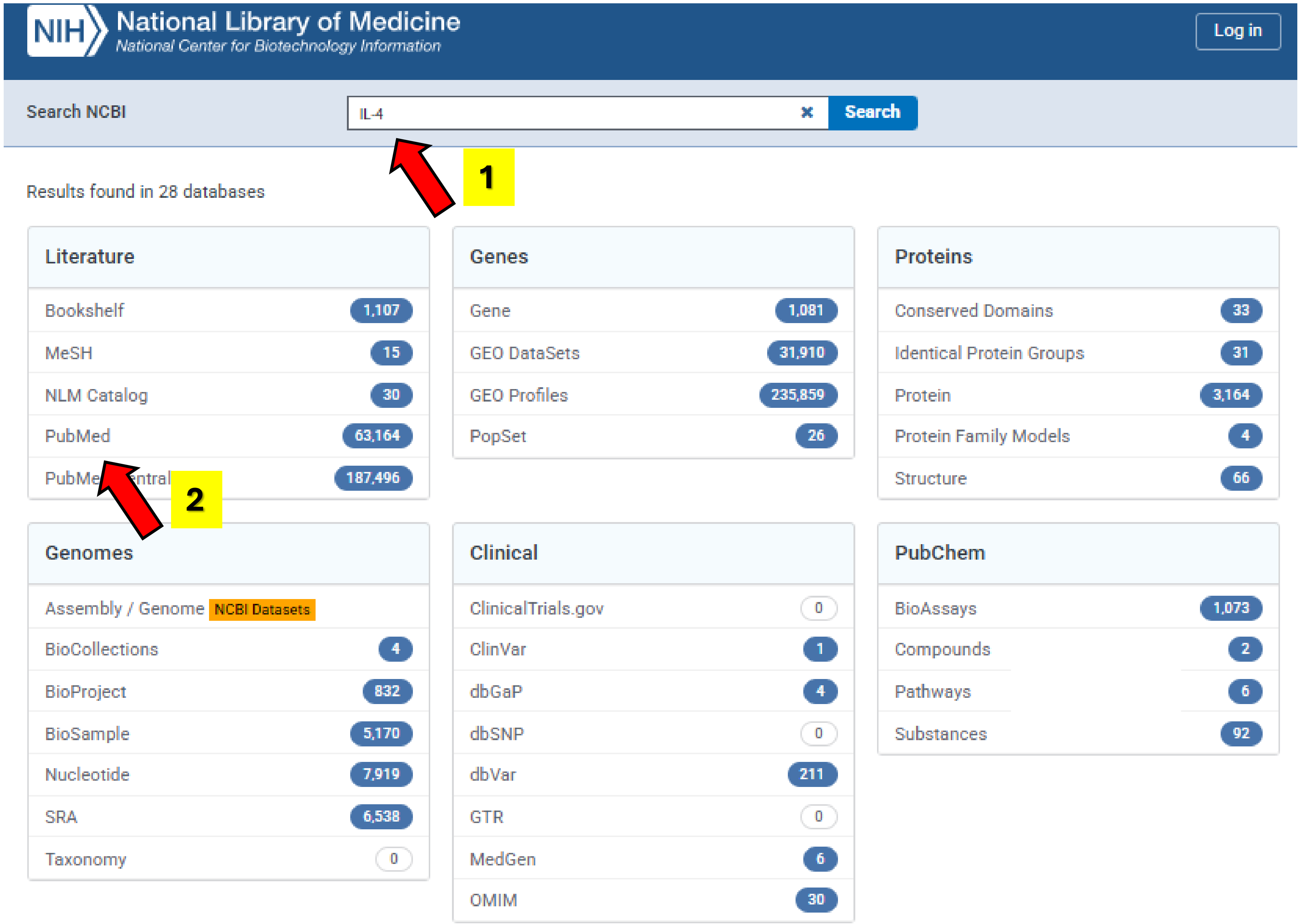
NIH
National Library of Medicine
National Center for Biotechnology Information
Log in
Search NCBI
IL-4
Search
1
Results found in 28 databases
Literature
Bookshelf 1,107
MeSH 15
NLM Catalog 30
PubMed 63,164
PubMe ntral 187,496
2
Genes
Gene 1,081
GEO DataSets 31,910
GEO Profiles 235,859
PopSet 26
Proteins
Conserved Domains 33
Identical Protein Groups 31
Protein 3,164
Protein Family Models 4
Structure 66
Genomes
Assembly / Genome NCBI Datasets
BioCollections 4
BioProject 832
BioSample 5,170
Nucleotide 7,919
SRA 6,538
Taxonomy 0
Clinical
ClinicalTrials.gov 0
ClinVar 1
dbGaP 4
dbSNP 0
dbVar 211
GTR 0
MedGen 6
OMIM 30
PubChem
BioAssays 1,073
Compounds 2
Pathways 6
Substances 92

STEP_01

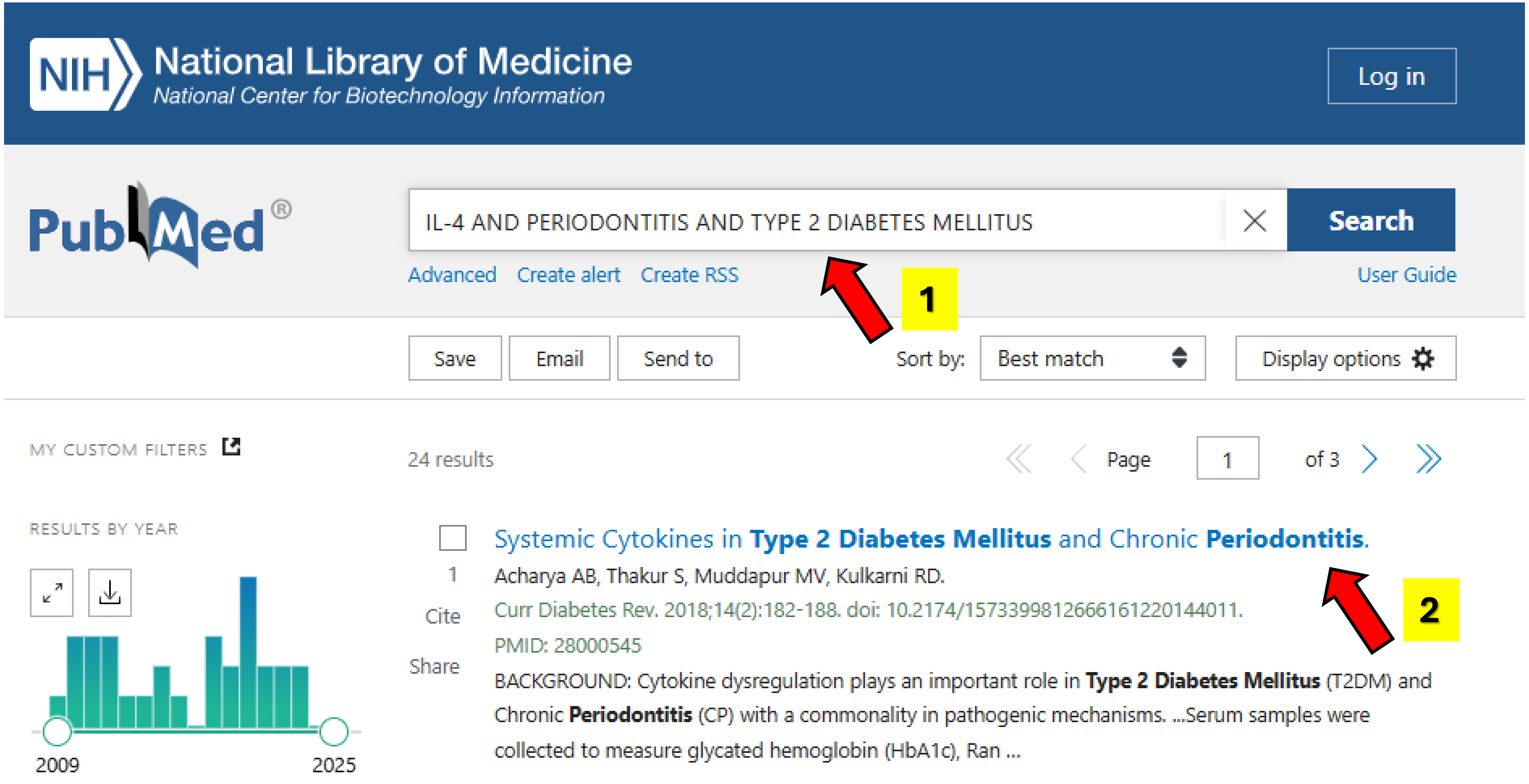
NIH
National Library of Medicine
National Center for Biotechnology Information
Log in
PubMed®
IL-4 AND PERIODONTITIS AND TYPE 2 DIABETES MELLITUS
Search
Advanced
Create alert
Create RSS
User Guide
1
Save
Email
Send to
Sort by:
Best match
Display options
MY CUSTOM FILTERS
24 results
Page
1
of 3
RESULTS BY YEAR
2009
2025
Systemic Cytokines in Type 2 Diabetes Mellitus and Chronic Periodontitis.
1
Cite
Share
Acharya AB, Thakur S, Muddapur MV, Kulkarni RD.
Curr Diabetes Rev. 2018;14(2):182-188. doi: 10.2174/1573399812666161220144011.
PMID: 28000545
BACKGROUND: Cytokine dysregulation plays an important role in Type 2 Diabetes Mellitus (T2DM) and Chronic Periodontitis (CP) with a commonality in pathogenic mechanisms. ...Serum samples were collected to measure glycated hemoglobin (HbA1c), Ran ...
2

STEP_02

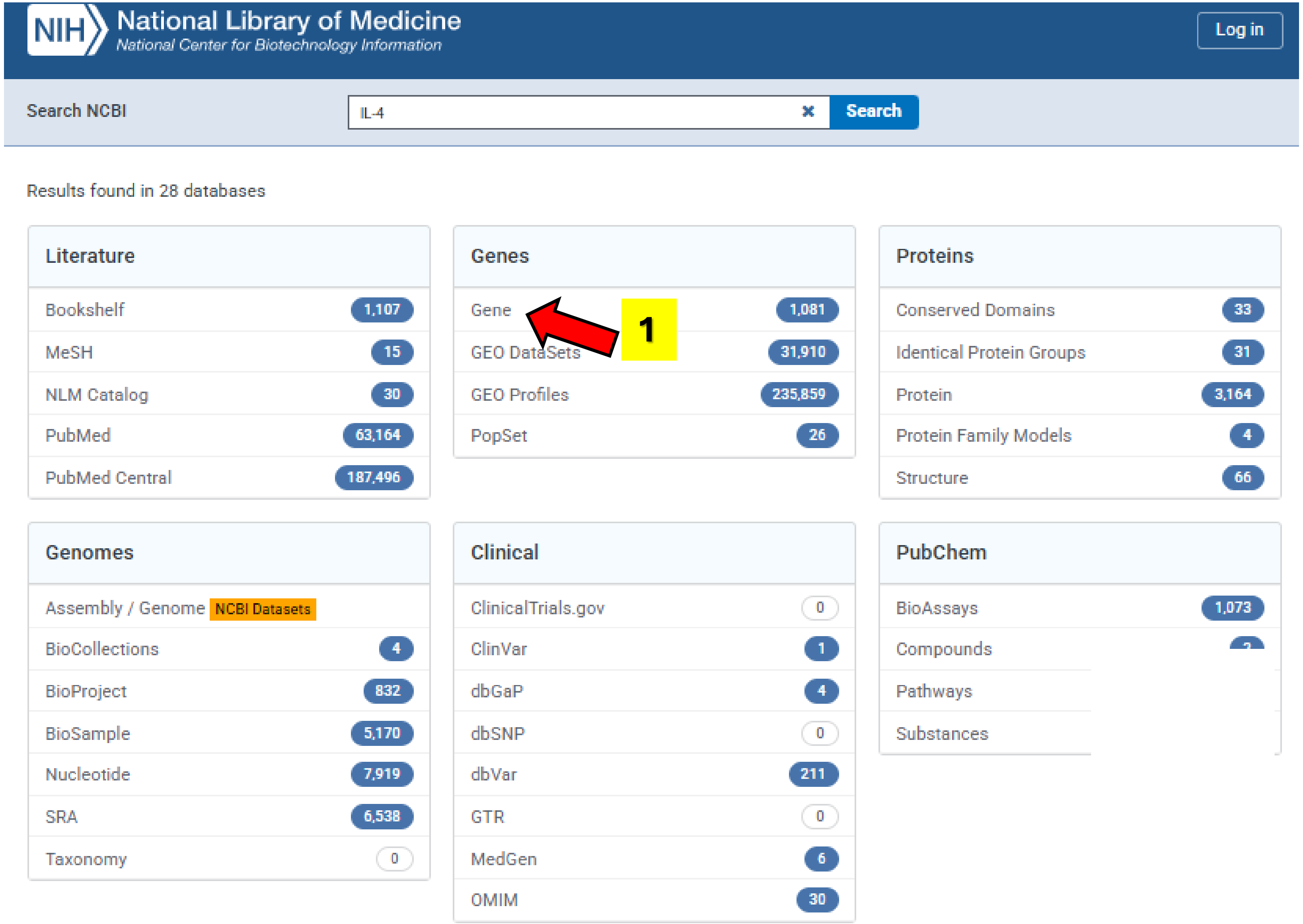

NIH
National Library of Medicine
National Center for Biotechnology Information
Log in
Search NCBI
IL-4
Search
Results found in 28 databases
Literature
Bookshelf 1,107
MeSH 15
NLM Catalog 30
PubMed 63,164
PubMed Central 187,496
Genes
Gene 1,081
GEO DataSets 31,910
GEO Profiles 235,859
PopSet 26
1
Proteins
Conserved Domains 33
Identical Protein Groups 31
Protein 3,164
Protein Family Models 4
Structure 66
Genomes
Assembly / Genome NCBI Datasets
BioCollections 4
BioProject 832
BioSample 5,170
Nucleotide 7,919
SRA 6,538
Taxonomy 0
Clinical
ClinicalTrials.gov 0
ClinVar 1
dbGaP 4
dbSNP 0
dbVar 211
GTR 0
MedGen 6
OMIM 30
PubChem
BioAssays 1,073
Compounds
Pathways
Substances

STEP_02

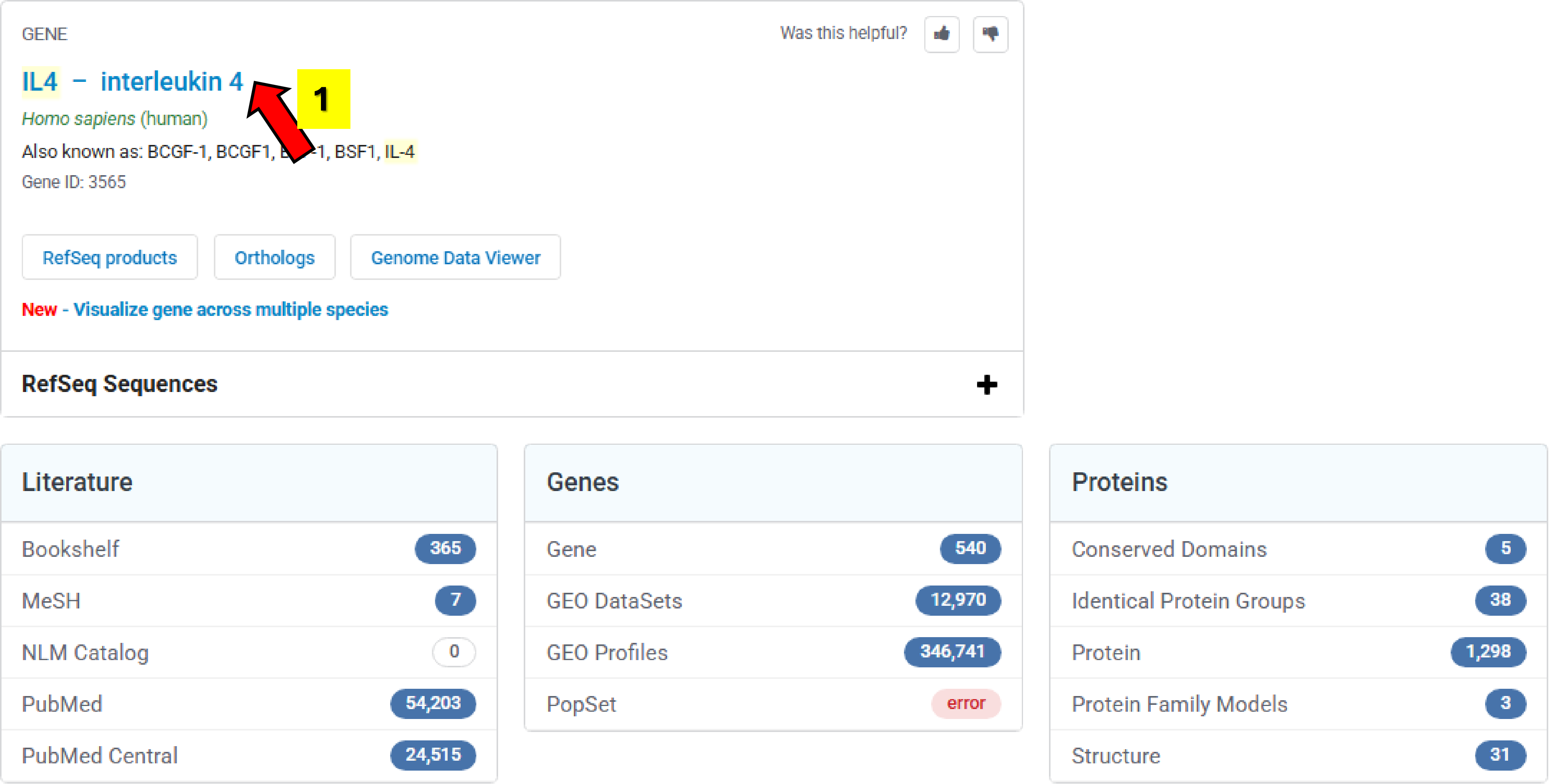
GENE
Was this helpful?
IL4 – interleukin 4
1
Homo sapiens (human)
Also known as: BCGF-1, BCGF1,
1, BSF1, IL-4
Gene ID: 3565
RefSeq products
Orthologs
Genome Data Viewer
New - Visualize gene across multiple species
RefSeq Sequences
Literature
Bookshelf 365
MeSH 7
NLM Catalog 0
PubMed 54,203
PubMed Central 24,515
Genes
Gene 540
GEO DataSets 12,970
GEO Profiles 346,741
PopSet error
Proteins
Conserved Domains 5
Identical Protein Groups 38
Protein 1,298
Protein Family Models 3
Structure 31

STEP_02

National Library of Medicine
National Center for Biotechnology Information

Gene | Gene | (il4[gene]) AND (Homo sapiens[orgn]) AND alive[prop] NOT newentry[gene]

Create RSS Save search Advanced

Full Report Send to:

Showing Current items.

1

IL4 interleukin 4 [*Homo sapiens* (human)]

Download Datasets

Gene ID: 3565, updated on 4-Jan-2025

Summary

**Official Symbol**	IL4 provided by HGNC
**Official Full Name**	interleukin 4 provided by HGNC
**Primary source**	HGNC:HGNC:6014
**See related**	Ensembl:ENSG00000113520 MIM:147780; AllianceGenome:HGNC:6014
**Gene type**	protein coding
**RefSeq status**	REVIEWED
**Organism**	Homo sapiens
**Lineage**	Eukaryota; Metazoa; Chordata; Craniata; Vertebrata; Euteleostomi; Mammalia; Eutheria; Euarchontoglires; Primates; Haplorrhini; Catarrhini; Hominidae; Homo
**Also known as**	BSF1; IL-4; BCGF1; BSF-1; BCGF-1
**Summary**	The protein encoded by this gene is a pleiotropic cytokine produced by activated T cells. This cytokine is a ligand for interleukin 4 receptor. The interleukin 4 receptor also binds to IL13, which may contribute to many overlapping functions of this cytokine and IL13. STAT6, a signal transducer and activator of transcription, has been shown to play a central role in mediating the immune regulatory signal of this cytokine. This gene, IL3, IL5, IL13, and CSF2 form a cytokine gene cluster on chromosome 5q, with this gene particularly close to IL13. This gene, IL13 and IL5 are found to be regulated coordinately by several long-range regulatory elements in an over 120 kilobase range on the chromosome. IL4 is considered an important cytokine for tissue repair, counterbalancing the effects of proinflammatory type 1 cytokines, however, it also promotes allergic airway inflammation. Moreover, IL-4, a type 2 cytokine, mediates and regulates a variety of human host responses such as allergic, anti-parasitic, wound healing, and acute inflammation. This cytokine has been reported to promote resolution of neutrophil-mediated acute lung injury. In an allergic response, IL-4 has an essential role in the production of allergen-specific immunoglobin (Ig) E. This pro-inflammatory cytokine has been observed to be increased in COVID-19 (Coronavirus disease 2019) patients, but is not necessarily associated with severe COVID-19 pathology. Two alternatively spliced transcript variants of this gene encoding distinct isoforms have been reported. [provided by RefSeq, Aug 2020]
**Annotation information**	Note: This gene has been reviewed for its involvement in coronavirus biology, and is relevant for disease process.
**Expression**	Low expression observed in reference dataset See more
**Orthologs**	mouse all

2

STEP_02

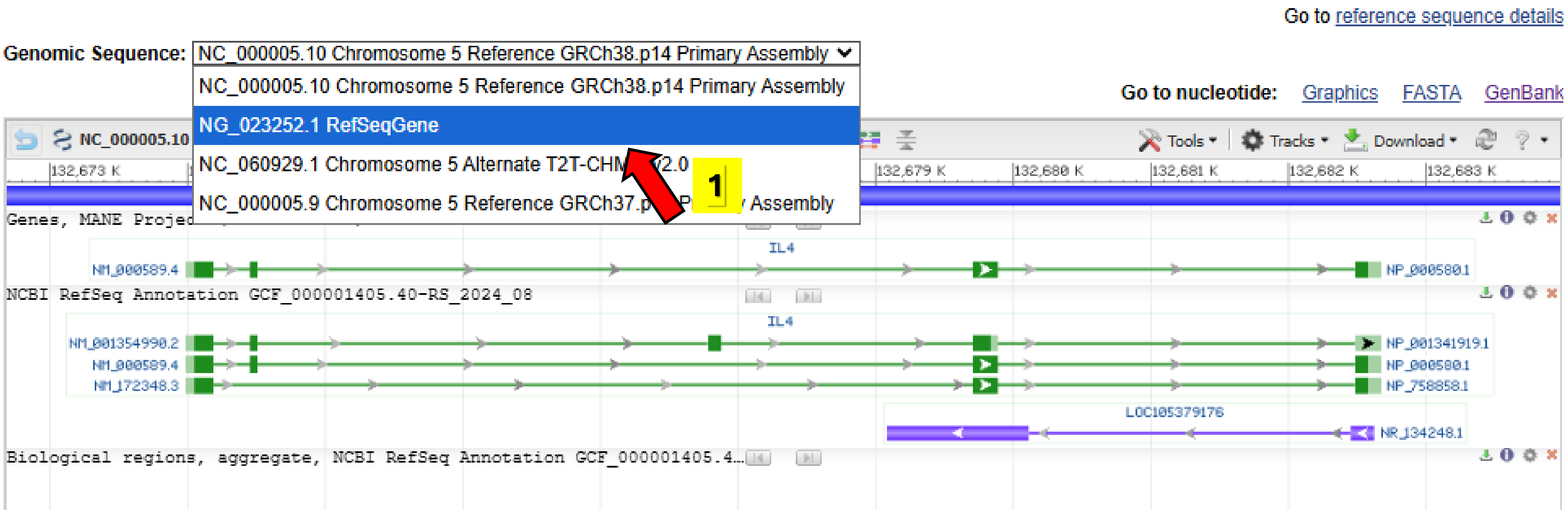
Go to reference sequence details
Genomic Sequence:
NC_000005.10 Chromosome 5 Reference GRCh38.p14 Primary Assembly
NC_000005.10 Chromosome 5 Reference GRCh38.p14 Primary Assembly
NG_023252.1 RefSeqGene
NC_060929.1 Chromosome 5 Alternate T2T-CHM v2.0
NC_000005.9 Chromosome 5 Reference GRCh37.p Assembly
1
Go to nucleotide:
Graphics
FASTA
GenBank
NC_000005.10
Tools
Tracks
Download
132,673 K
132,679 K
132,680 K
132,681 K
132,682 K
132,683 K
Genes, MANE Projec
IL4
NM_000589.4
NP_000580.1
NCBI RefSeq Annotation GCF_000001405.40-RS_2024_08
IL4
NM_001354990.2
NP_001341919.1
NM_000589.4
NP_000580.1
NM_172348.3
NP_758858.1
LOC105379176
NR_134248.1
Biological regions, aggregate, NCBI RefSeq Annotation GCF_000001405.4...

STEP_02

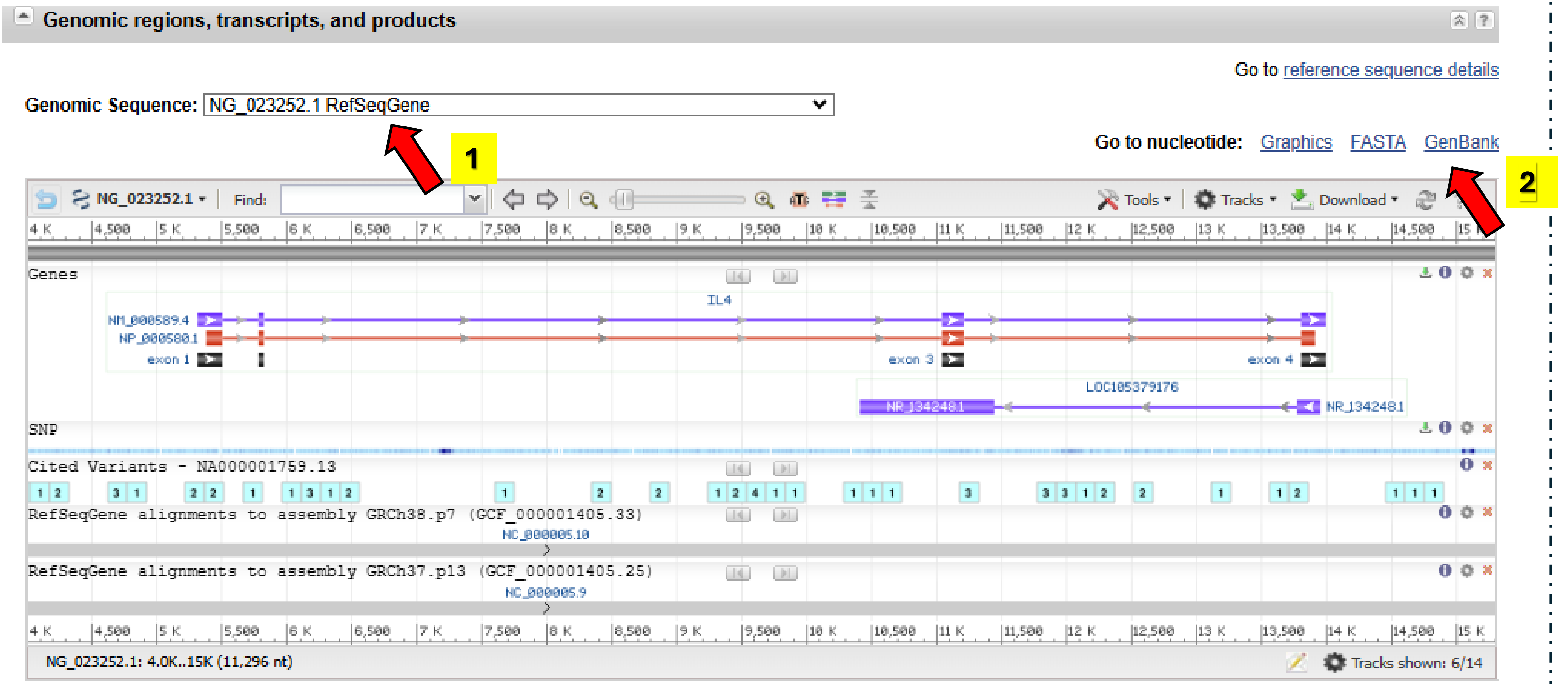

Genomic regions, transcripts, and products
Go to reference sequence details
Genomic Sequence: NG_023252.1 RefSeqGene
1
Go to nucleotide: Graphics FASTA GenBank
2
NG_023252.1
Find:
Tools
Tracks
Download
Genes
IL4
NM_000589.4
NP_000580.1
exon 1
exon 3
exon 4
LOC105379176
NR_134248.1
SNP
Cited Variants - NA000001759.13
RefSeqGene alignments to assembly GRCh38.p7 (GCF_000001405.33)
NC_000005.10
RefSeqGene alignments to assembly GRCh37.p13 (GCF_000001405.25)
NC_000005.9
NG_023252.1: 4.0K..15K (11,296 nt)
Tracks shown: 6/14

STEP_02

An official website of the United States government Here's how you know

NIH National Library of Medicine
National Center for Biotechnology Information

Nucleotide | Nucleotide
Advanced

GenBank | Send to:

Homo sapiens chromosome 5, GRCh38.p14 Primary Assembly

1

NCBI Reference Sequence: NC_000005.10

FASTA Graphics

```
LOCUS       NC_000005               8690 bp    DNA     linear   CON 26-AUG-2024
DEFINITION  Homo sapiens chromosome 5, GRCh38.p14 Primary Assembly.
ACCESSION   NC_000005 REGION: 132673989..132682678
VERSION     NC_000005.10
DBLINK      BioProject: PRJNA168
            Assembly: GCF_000001405.40
KEYWORDS    RefSeq.
SOURCE      Homo sapiens (human)
  ORGANISM  Homo sapiens
                 ##Genome-Annotation-Data-END##
FEATURES             Location/Qualifiers
     source          1..8690
                     /organism="Homo sapiens"
                     /mol_type="genomic DNA"
                     /db_xref="taxon:9606"
                     /chromosome="5"
     gene            1..8690
                     /gene="IL4"
                     /gene_synonym="BCGF-1; BCGF1; BSF-1; BSF1; IL-4"
                     /note="interleukin 4; Derived by automated computational
                     analysis using gene prediction method: BestRefSeq."
                     /db_xref="GeneID:3565"
                     /db_xref="HGNC:HGNC:6014"
                     /db_xref="MIM:147780"
     mRNA            join(1..197,471..518,3793..3893,5726..5902,8498..8690)
                     /gene="IL4"
                     /gene_synonym="BCGF-1; BCGF1; BSF-1; BSF1; IL-4"
                     /product="interleukin 4, transcript variant 3"
                     /note="Derived by automated computational analysis using
                     gene prediction method: BestRefSeq."
                     /transcript_id="NM_001354990.2"
                     /db_xref="GeneID:3565"
                     /db_xref="HGNC:HGNC:6014"
                     /db_xref="MIM:147780"
```

2

3

```
ORIGIN
        1 atcgttagct tctcctgata aactaattgc ctcacattgt cactgcaaat cgacacctat
       61 taatgggtct cacctcccaa ctgcttcccc ctctgttctt cctgctagca tgtgccggca
      121 actttgtcca cggacacaag tgcgatatca ccttacagga gatcatcaaa actttgaaca
      181 gcctcacaga gcagaaggtg agtacctatc tggcaccatc tctccagatg ttctggtgat
      241 gctctcagta tttctaggca tgaaaacgtt aacagctgct agagaagttg gaactggtgg
      301 ttggtggcag tccagggcac acagcgaggc ttctcccctg ccactctttt ttctgagggt
      361 ttgtaggaag tttcctcagt tggagggagt gagagctgct catcaaggac ttctctgtcc
      421 ggttggaggt taactctgtc tcttgctctc tcatttctgc ctggaccaag actctgtgca
      481 ccgagttgac cgtaacagac atctttgctg cctccaaggt aagaagccgt cccacggtct
      541 gttttagcaa atggggagat ccatccccaa atgtctgaac aagaaacttg tctaatggaa
      601 aacgagcggg cccaaattaa ctctaaggtg ttagatgttt tcaaagaacg agaagtctga
      661 tctttactct taagcatgtt ttggtctttc tggtttcact tgatttagaa gacatgtaat
      721 agaaagctta catgctgtag tcctgactca gatcctggtc aaagaaaagc cctcttgggt
      781 tttacttagc tttggcatag tgcctggaac gtaggaggca ctcaataaat gcctgttgaa
      841 tgagagaatt tttctggccc atacatttct gaaaaaccaa atactctcac agaaacagat
      901 attgagatga caggttgagg gagctttcat tttgtctaag agacttccta tggcaacaga
      961 aaaggtatcg ccagagcccc tcctcttcca cagcctggcc acctaacagc cctctgggtt
     1021 ccggggctgg ccgtccagag ctcctcagct tgctctggcc ggccgaactc ccctccagct
     1081 cggtctggaa ccatcctgct gggcagcgtc cagcacatcc ctgcttcggg ctgcctgggc
     1141 acctcgcctc tctgcctcct gtgctgcctc acccccaccc ctctatctgt agtgggagga
     1201 gatagatttg acagctgata gtgcattttc tctgacaaac acatgactac agccgtatca
     1261 atagttttgt gcatttcagt tcctgttttc atggaaacac acggctgaga atgaaagccc
     1321 caaagcctca atttcacagt ggtctcctaa ctacctgctt tccatgcaaa ctagggagat
     1381 gatatggcca ggagtgaagc cctgtgtgtt gggcagggtc acactccagc acccagacca
     1441 tagaacaggg cccatcctgc ttcatgaggg aaactgctct tcgggccttt agctggacta
     1501 tctcatttca ttagttatcc cgggagtccg atacaggatg agattctgaa gggcaaatac
     1561 acactttttt tttttttga gatagggtct tgttctgtca cccaggctgg agtgcagtgg
     1621 tgcgatttca gctcatagca gcctccacct cccaggctca agctatcttc ctacctcagc
     1681 ctcccaagta gccgggacga caggtgtgca ccaccacgcc tggctaattt ttgtattttt
     1741 ttgtagagat ggagtcttgc cattttgccc aggcttgtct cgaacttctg ggctcaagca
     1801 atccgtccac ctcggcctct caaagtgctg ggattagcca ctgcacctgg gcaacagttt
     1861 atgtgtgtgt gtgtgtgtgt gtgtgtgtgt atatatgtgt gtgtgtatat atatgtgtgt
     1921 atgtatatat gtgtatgtat atgtgtgtgt gtgtgtgtgt gtgtgtgtat aaaatctcca
     1981 agtccatcca accgagatgg ctcctactag aagccaagag tccaccgggt tgagcactgg
     2041 gtctctggag gcctgtggca ctgctgagaa ggctctaaca aagccaaggg aagggccacc
     2101 tcactagaag ccaggcctgg aggaagggtg agggctgagg gcctggaggt aagactgcct
```

STEP_03

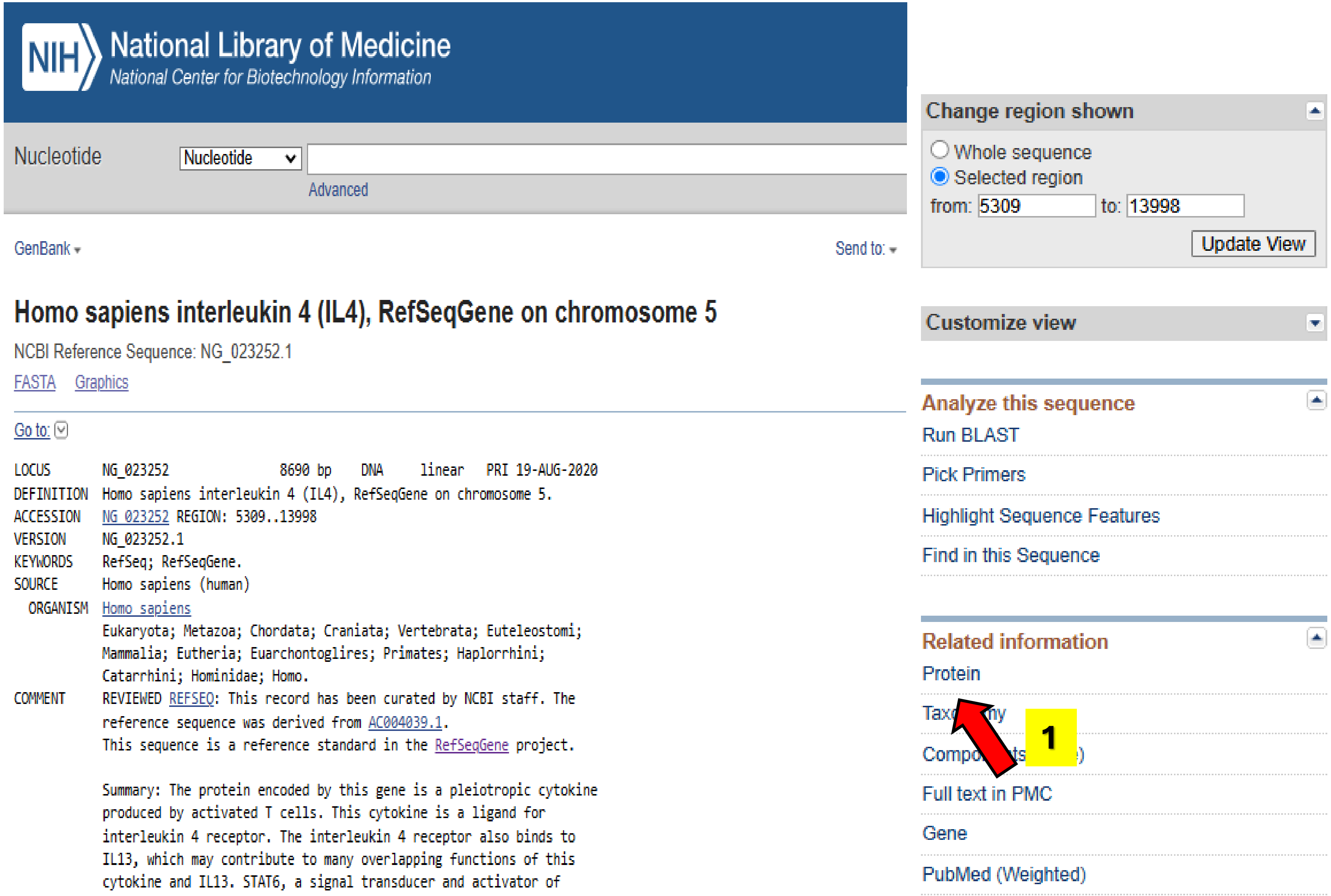
NIH
National Library of Medicine
National Center for Biotechnology Information
Nucleotide
Nucleotide
Advanced
GenBank
Send to:
Homo sapiens interleukin 4 (IL4), RefSeqGene on chromosome 5
NCBI Reference Sequence: NG_023252.1
FASTA Graphics
Go to:
LOCUS NG_023252 8690 bp DNA linear PRI 19-AUG-2020
DEFINITION Homo sapiens interleukin 4 (IL4), RefSeqGene on chromosome 5.
ACCESSION NG_023252 REGION: 5309..13998
VERSION NG_023252.1
KEYWORDS RefSeq; RefSeqGene.
SOURCE Homo sapiens (human)
ORGANISM Homo sapiens
Eukaryota; Metazoa; Chordata; Craniata; Vertebrata; Euteleostomi;
Mammalia; Eutheria; Euarchontoglires; Primates; Haplorrhini;
Catarrhini; Hominidae; Homo.
COMMENT REVIEWED REFSEQ: This record has been curated by NCBI staff. The
reference sequence was derived from AC004039.1.
This sequence is a reference standard in the RefSeqGene project.
Summary: The protein encoded by this gene is a pleiotropic cytokine
produced by activated T cells. This cytokine is a ligand for
interleukin 4 receptor. The interleukin 4 receptor also binds to
IL13, which may contribute to many overlapping functions of this
cytokine and IL13. STAT6, a signal transducer and activator of
Change region shown
Whole sequence
Selected region
from: 5309 to: 13998
Update View
Customize view
Analyze this sequence
Run BLAST
Pick Primers
Highlight Sequence Features
Find in this Sequence
Related information
Protein
1
Full text in PMC
Gene
PubMed (Weighted)

STEP_03

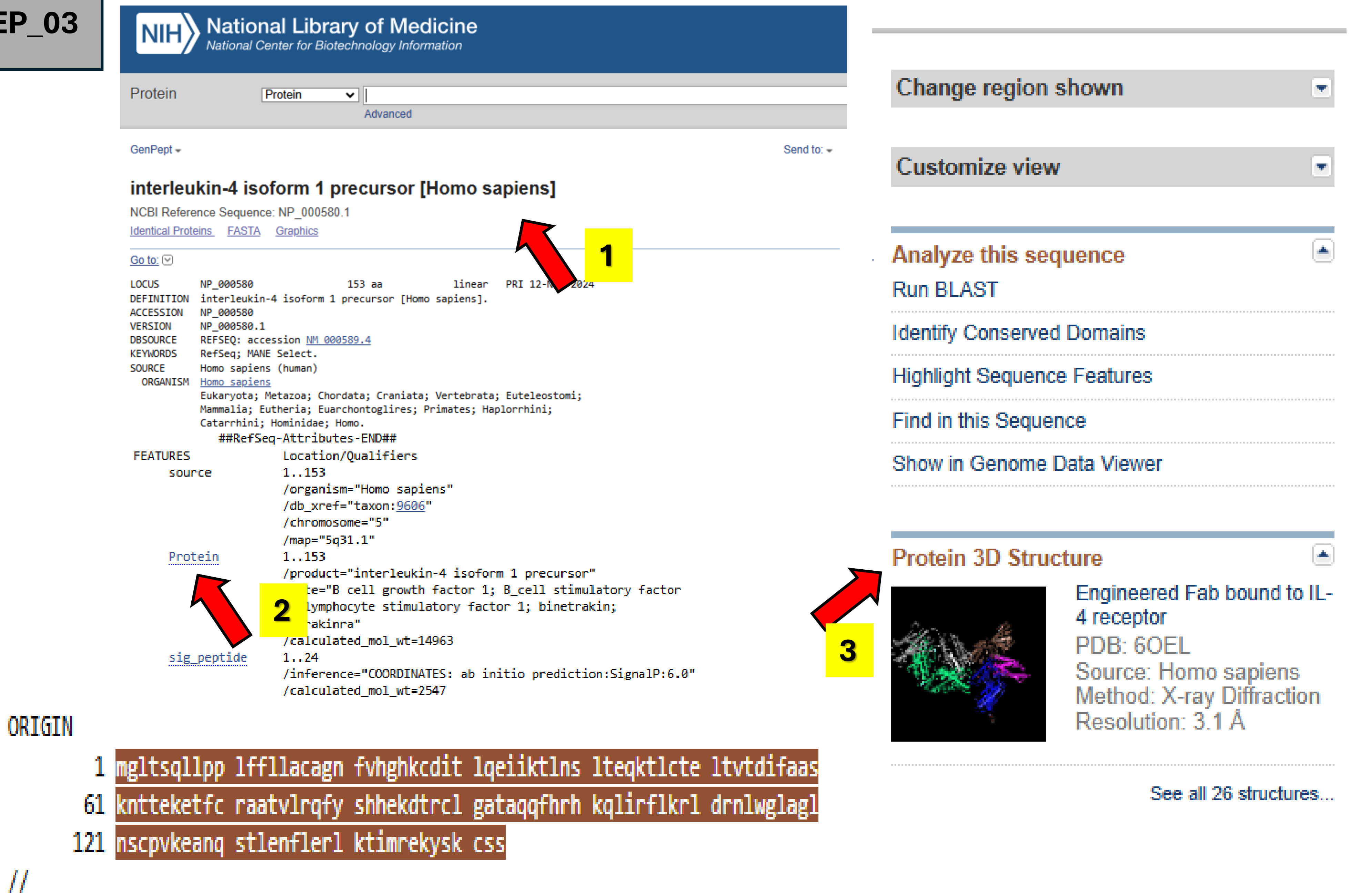
NIH National Library of Medicine
National Center for Biotechnology Information
Protein
Advanced
GenPept
Send to:
interleukin-4 isoform 1 precursor [Homo sapiens]
NCBI Reference Sequence: NP_000580.1
Identical Proteins FASTA Graphics
Go to:
LOCUS NP_000580 153 aa linear PRI
DEFINITION interleukin-4 isoform 1 precursor [Homo sapiens].
ACCESSION NP_000580
VERSION NP_000580.1
DBSOURCE REFSEQ: accession NM_000589.4
KEYWORDS RefSeq; MANE Select.
SOURCE Homo sapiens (human)
ORGANISM Homo sapiens
Eukaryota; Metazoa; Chordata; Craniata; Vertebrata; Euteleostomi;
Mammalia; Eutheria; Euarchontoglires; Primates; Haplorrhini;
Catarrhini; Hominidae; Homo.
##RefSeq-Attributes-END##
FEATURES Location/Qualifiers
source 1..153
/organism="Homo sapiens"
/db_xref="taxon:9606"
/chromosome="5"
/map="5q31.1"
Protein 1..153
/product="interleukin-4 isoform 1 precursor"
B cell growth factor 1; B_cell stimulatory factor
lymphocyte stimulatory factor 1; binetrakin;
/calculated_mol_wt=14963
sig_peptide 1..24
/inference="COORDINATES: ab initio prediction:SignalP:6.0"
/calculated_mol_wt=2547
ORIGIN
1 mgltsqllpp lffllacagn fvhghkcdit lqeiiktlns lteqktlcte ltvtdifaas
61 kntteketfc raatvlrqfy shhekdtrcl gataqqfhrh kqlirflkrl drnlwglagl
121 nscpvkeanq stlenflerl ktimrekysk css
//
Change region shown
Customize view
Analyze this sequence
Run BLAST
Identify Conserved Domains
Highlight Sequence Features
Find in this Sequence
Show in Genome Data Viewer
Protein 3D Structure
Engineered Fab bound to IL-4 receptor
PDB: 6OEL
Source: Homo sapiens
Method: X-ray Diffraction
Resolution: 3.1 Å
See all 26 structures...
1
2
3

STEP_03

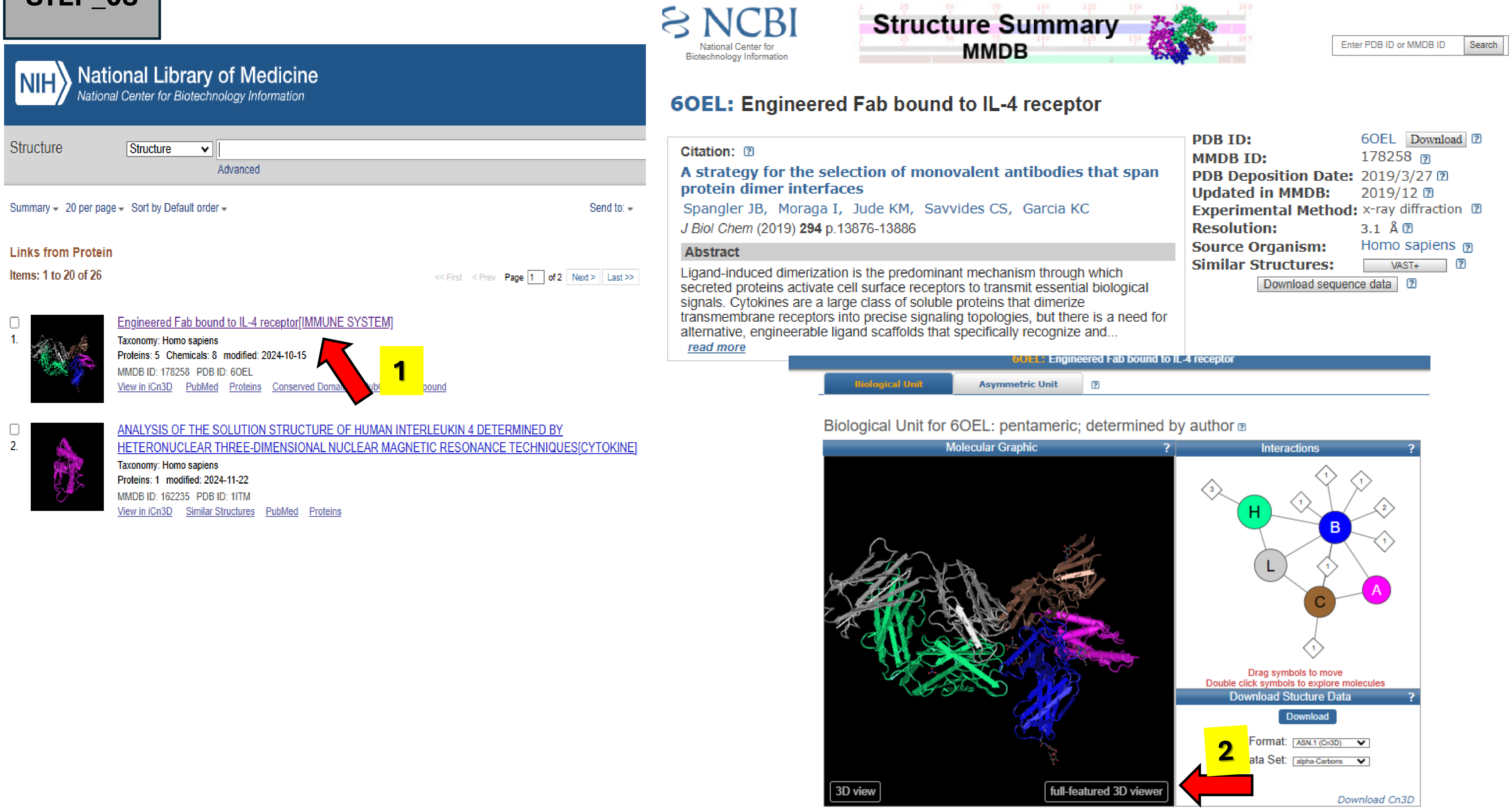

National Library of Medicine
National Center for Biotechnology Information
Structure
Advanced
Summary 20 per page Sort by Default order
Send to:
Links from Protein
Items: 1 to 20 of 26
<< First < Prev Page 1 of 2 Next > Last >>
Engineered Fab bound to IL-4 receptor[IMMUNE SYSTEM]
Taxonomy: Homo sapiens
Proteins: 5 Chemicals: 8 modified: 2024-10-15
MMDB ID: 178258 PDB ID: 6OEL
View in iCn3D PubMed Proteins
1
ANALYSIS OF THE SOLUTION STRUCTURE OF HUMAN INTERLEUKIN 4 DETERMINED BY HETERONUCLEAR THREE-DIMENSIONAL NUCLEAR MAGNETIC RESONANCE TECHNIQUES[CYTOKINE]
Taxonomy: Homo sapiens
Proteins: 1 modified: 2024-11-22
MMDB ID: 162235 PDB ID: 1ITM
View in iCn3D Similar Structures PubMed Proteins
NCBI
National Center for Biotechnology Information
Structure Summary
MMDB
Enter PDB ID or MMDB ID
Search
6OEL: Engineered Fab bound to IL-4 receptor
Citation:
A strategy for the selection of monovalent antibodies that span protein dimer interfaces
Spangler JB, Moraga I, Jude KM, Savvides CS, Garcia KC
J Biol Chem (2019) 294 p.13876-13886
Abstract
Ligand-induced dimerization is the predominant mechanism through which secreted proteins activate cell surface receptors to transmit essential biological signals. Cytokines are a large class of soluble proteins that dimerize transmembrane receptors into precise signaling topologies, but there is a need for alternative, engineerable ligand scaffolds that specifically recognize and...
read more
PDB ID: 6OEL Download
MMDB ID: 178258
PDB Deposition Date: 2019/3/27
Updated in MMDB: 2019/12
Experimental Method: x-ray diffraction
Resolution: 3.1 Å
Source Organism: Homo sapiens
Similar Structures: VAST+
Download sequence data
6OEL: Engineered Fab bound to IL-4 receptor
Biological Unit
Asymmetric Unit
Biological Unit for 6OEL: pentameric; determined by author
Molecular Graphic
Interactions
H
B
L
A
C
Drag symbols to move
Double click symbols to explore molecules
Download Stucture Data
Download
Format: ASN.1 (Cn3D)
ata Set: alpha-Carbons
Download Cn3D
3D view
full-featured 3D viewer
2

STEP_03

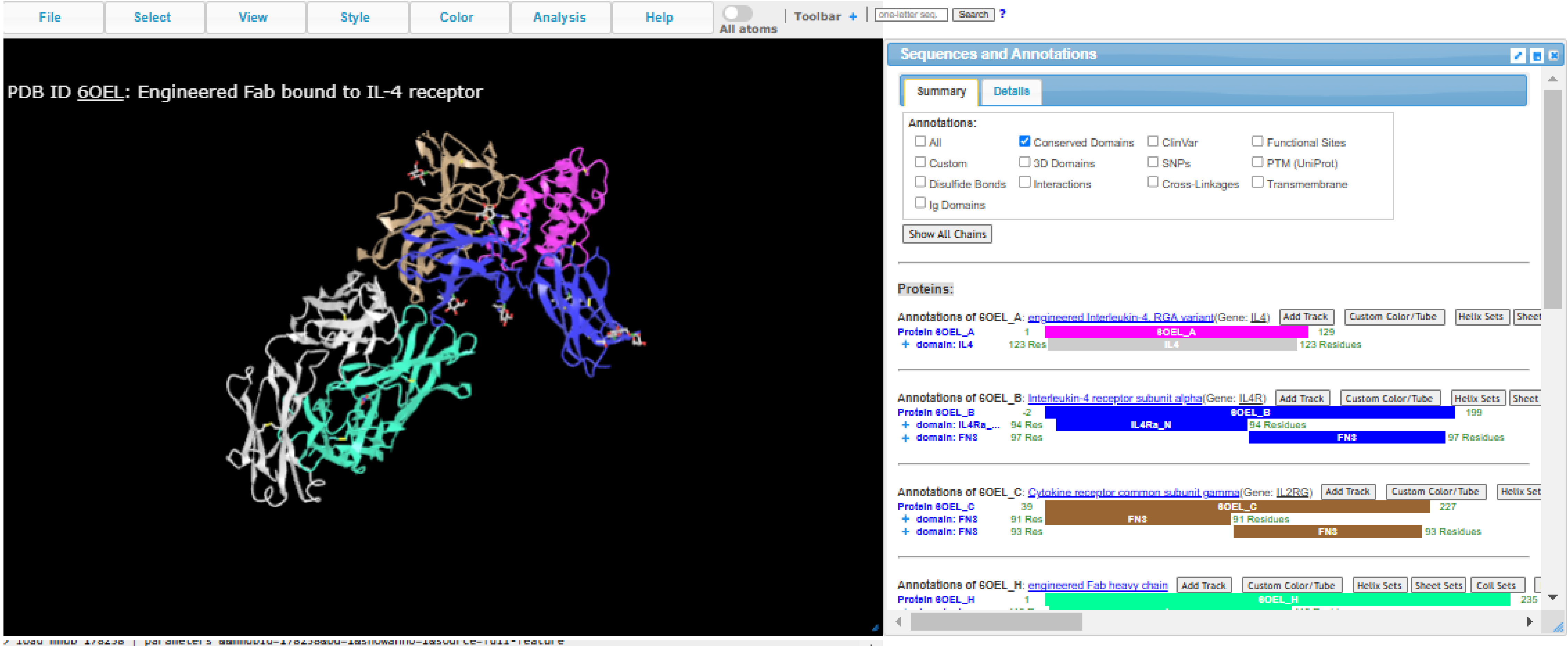
File
Select
View
Style
Color
Analysis
Help
All atoms
Toolbar +
Search
PDB ID 6OEL: Engineered Fab bound to IL-4 receptor
Sequences and Annotations
Summary
Details
Annotations:
All
Conserved Domains
ClinVar
Functional Sites
Custom
3D Domains
SNPs
PTM (UniProt)
Disulfide Bonds
Interactions
Cross-Linkages
Transmembrane
Ig Domains
Show All Chains
Proteins:
Annotations of 6OEL_A: engineered Interleukin-4, RGA variant(Gene: IL4)
Annotations of 6OEL_B: Interleukin-4 receptor subunit alpha(Gene: IL4R)
Annotations of 6OEL_C: Cytokine receptor common subunit gamma(Gene: IL2RG)
Annotations of 6OEL_H: engineered Fab heavy chain
Add Track
Custom Color/Tube
Helix Sets
Sheet Sets
Coil Sets

STEP_04

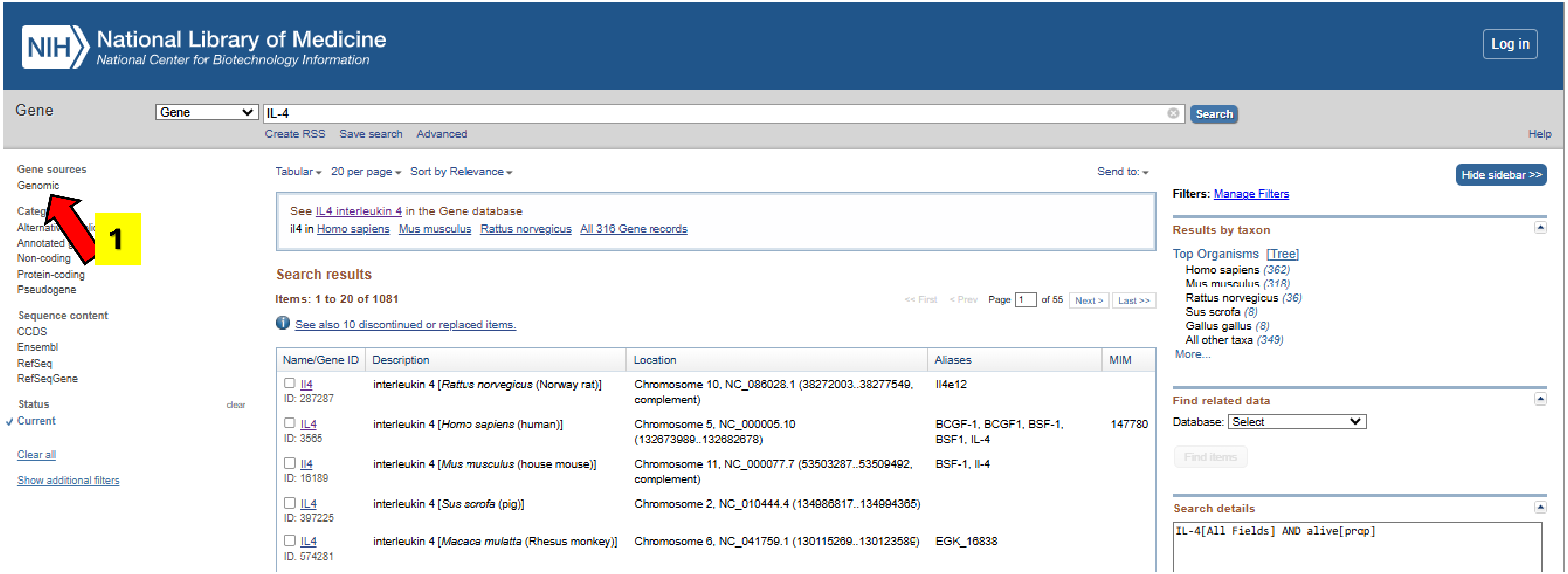

NIH
National Library of Medicine
National Center for Biotechnology Information
Log in
Gene
Gene
IL-4
Search
Create RSS
Save search
Advanced
Help
Gene sources
Genomic
1
Categ
Alternativ
Annotated
Non-coding
Protein-coding
Pseudogene
Sequence content
CCDS
Ensembl
RefSeq
RefSeqGene
Status
clear
Current
Clear all
Show additional filters
Tabular
20 per page
Sort by Relevance
Send to:
See IL4 interleukin 4 in the Gene database
il4 in Homo sapiens Mus musculus Rattus norvegicus All 316 Gene records
Search results
Items: 1 to 20 of 1081
<< First < Prev Page 1 of 55 Next > Last >>
See also 10 discontinued or replaced items.
Name/Gene ID
Description
Location
Aliases
MIM
Il4 ID: 287287
interleukin 4 [Rattus norvegicus (Norway rat)]
Chromosome 10, NC_086028.1 (38272003..38277549, complement)
Il4e12
IL4 ID: 3565
interleukin 4 [Homo sapiens (human)]
Chromosome 5, NC_000005.10 (132673989..132682678)
BCGF-1, BCGF1, BSF-1, BSF1, IL-4
147780
Il4 ID: 16189
interleukin 4 [Mus musculus (house mouse)]
Chromosome 11, NC_000077.7 (53503287..53509492, complement)
BSF-1, Il-4
IL4 ID: 397225
interleukin 4 [Sus scrofa (pig)]
Chromosome 2, NC_010444.4 (134988817..134994365)
IL4 ID: 574281
interleukin 4 [Macaca mulatta (Rhesus monkey)]
Chromosome 6, NC_041759.1 (130115269..130123589)
EGK_16838
Hide sidebar >>
Filters: Manage Filters
Results by taxon
Top Organisms [Tree]
Homo sapiens (362)
Mus musculus (318)
Rattus norvegicus (36)
Sus scrofa (8)
Gallus gallus (8)
All other taxa (349)
More...
Find related data
Database: Select
Find items
Search details
IL-4[All Fields] AND alive[prop]

STEP_04

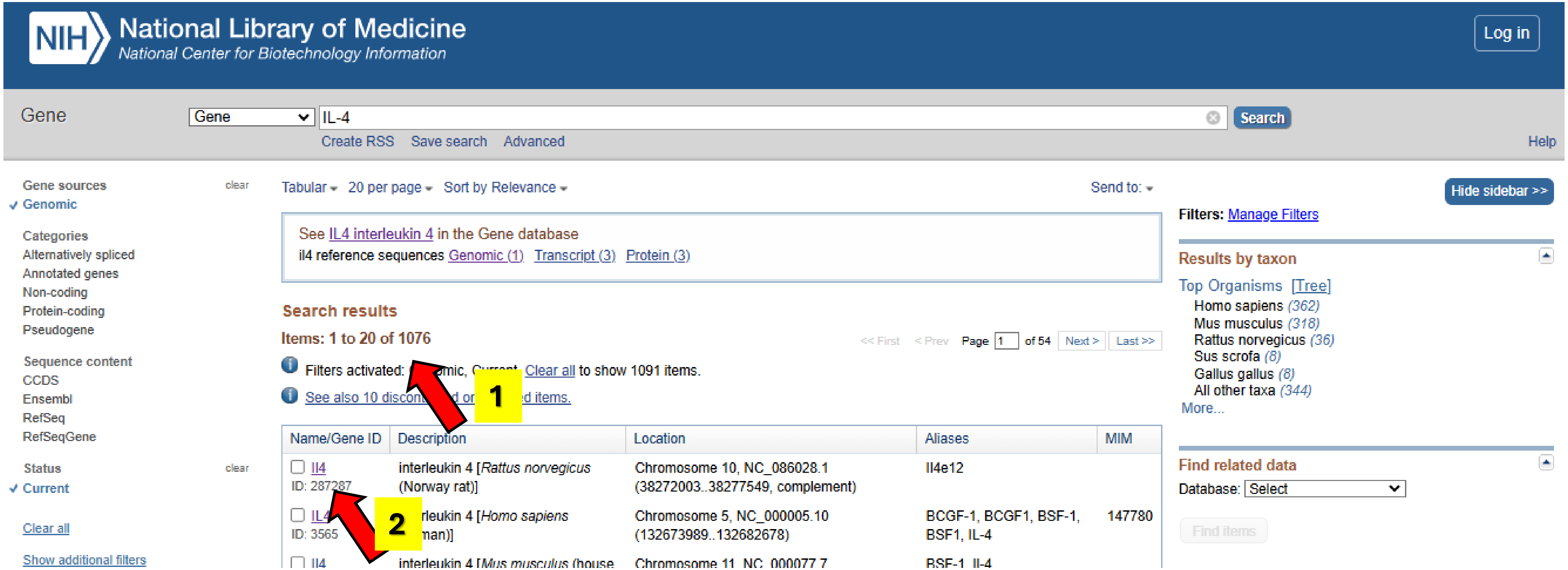
NIH
National Library of Medicine
National Center for Biotechnology Information
Log in
Gene
Gene
IL-4
Search
Create RSS
Save search
Advanced
Help
Gene sources
clear
Genomic
Categories
Alternatively spliced
Annotated genes
Non-coding
Protein-coding
Pseudogene
Sequence content
CCDS
Ensembl
RefSeq
RefSeqGene
Status
clear
Current
Clear all
Show additional filters
Tabular
20 per page
Sort by Relevance
Send to:
See IL4 interleukin 4 in the Gene database
il4 reference sequences Genomic (1) Transcript (3) Protein (3)
Search results
Items: 1 to 20 of 1076
<< First
< Prev
Page 1 of 54
Next >
Last >>
Clear all to show 1091 items.
1
2
Name/Gene ID
Description
Location
Aliases
MIM
Il4
ID: 287287
interleukin 4 [Rattus norvegicus (Norway rat)]
Chromosome 10, NC_086028.1 (38272003..38277549, complement)
Il4e12
ID: 3565
Chromosome 5, NC_000005.10 (132673989..132682678)
BCGF-1, BCGF1, BSF-1, BSF1, IL-4
147780
Hide sidebar >>
Filters: Manage Filters
Results by taxon
Top Organisms [Tree]
Homo sapiens (362)
Mus musculus (318)
Rattus norvegicus (36)
Sus scrofa (8)
Gallus gallus (8)
All other taxa (344)
More...
Find related data
Database: Select
Find items

STEP_04

IL4 interleukin 4 [*Homo sapiens* (human)]

Gene ID: 3565, updated on 4-Jan-2025

Download Datasets

Summary

Official Symbol IL4 provided by HGNC
Official Full Name interleukin 4 provided by HGNC
Primary source HGNC:HGNC:6014
See related Ensembl:ENSG00000113520 MIM:147780; AllianceGenome:HGNC:6014
Gene type protein coding
RefSeq status REVIEWED
Organism Homo sapiens
Lineage Eukaryota; Metazoa; Chordata; Craniata; Vertebrata; Euteleostomi; Mammalia; Eutheria; Euarchontoglires; Primates; Haplorrhini; Catarrhini; Hominidae; Homo
Also known as BSF1; IL-4; BCGF1; BSF-1; BCGF-1
Summary The protein encoded by this gene is a pleiotropic cytokine produced by activated T cells. This cytokine is a ligand for interleukin 4 receptor. The interleukin 4 receptor also binds to IL13, which may contribute to many overlapping functions of this cytokine and IL13. STAT6, a signal transducer and activator of transcription, has been shown to play a central role in mediating the immune regulatory signal of this cytokine. This gene, IL3, IL5, IL13, and CSF2 form a

Genomic context

Location: 5q31.1

See IL4 in Genome Data Viewer

Exon count: 5

Annotation release	Status	Assembly	Chr	Location
RS_2024_08	current	GRCh38.p14 (GCF_000001405.40)	5	NC_000005.10 (132673989..132682678)
RS_2024_08	current	T2T-CHM13v2.0 (GCF_009914755.1)	5	NC_060929.1 (133193788..133202478)
RS_2024_09	previous assembly	GRCh37.p13 (GCF_000001405.25)	5	NC_000005.9 (132009681..132018370)

Chromosome 5 - NC_000005.10

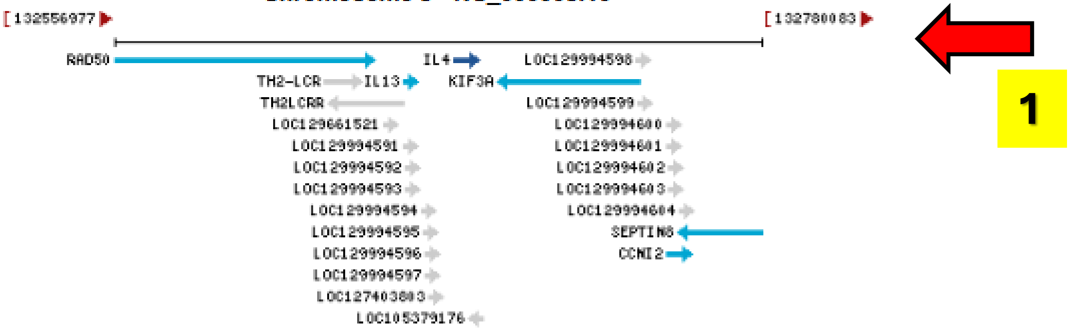

STEP_04

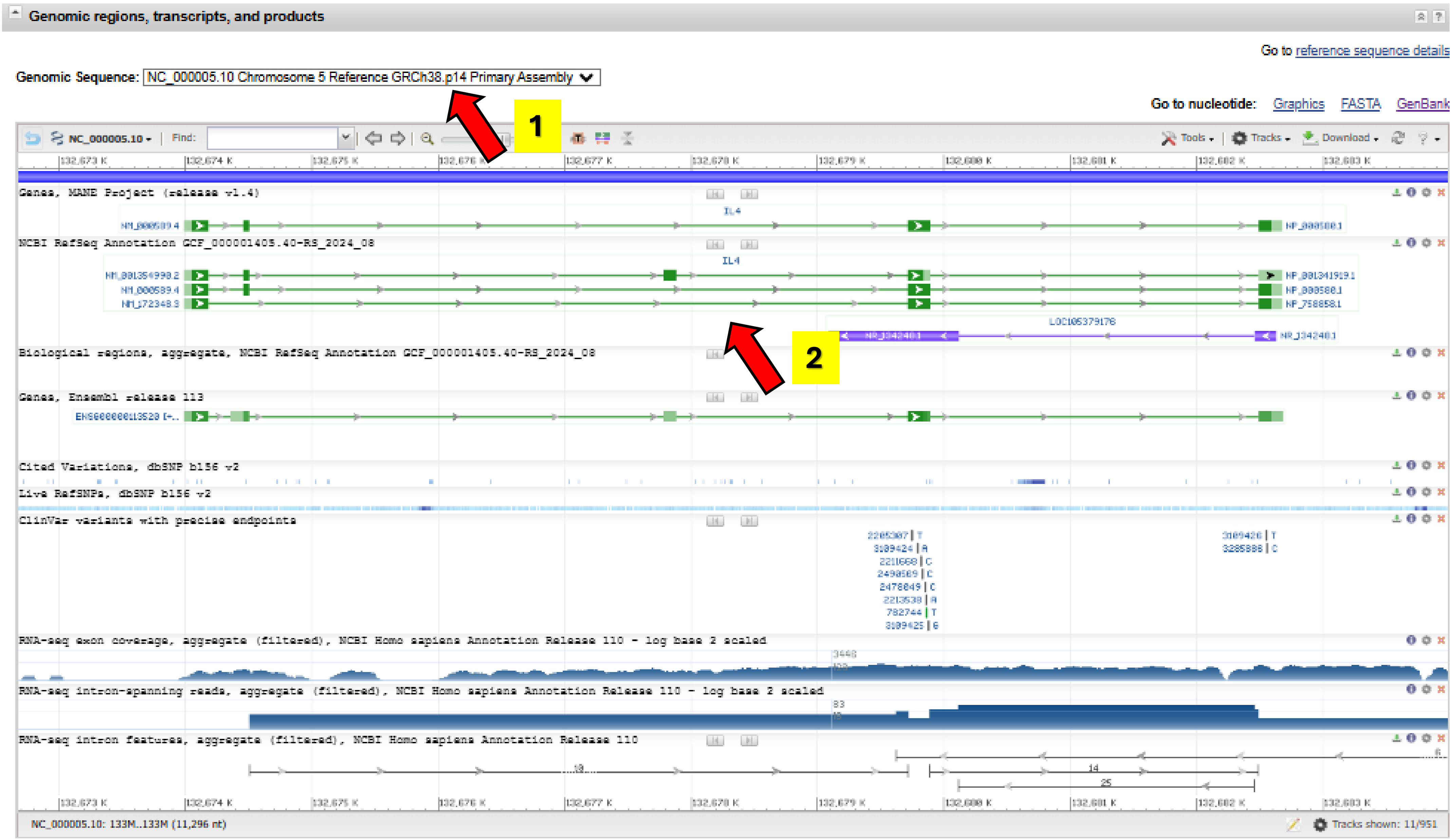

Genomic regions, transcripts, and products
Go to reference sequence details
Genomic Sequence: NC_000005.10 Chromosome 5 Reference GRCh38.p14 Primary Assembly
Go to nucleotide: Graphics FASTA GenBank
1
2
NC_000005.10
Find:
Tools
Tracks
Download
Genes, MANE Project (release v1.4)
IL4
NCBI RefSeq Annotation GCF_000001405.40-RS_2024_08
LOC105379176
Biological regions, aggregate, NCBI RefSeq Annotation GCF_000001405.40-RS_2024_08
Genes, Ensembl release 113
Cited Variations, dbSNP b156 v2
Live RefSNPs, dbSNP b156 v2
ClinVar variants with precise endpoints
RNA-seq exon coverage, aggregate (filtered), NCBI Homo sapiens Annotation Release 110 - log base 2 scaled
RNA-seq intron-spanning reads, aggregate (filtered), NCBI Homo sapiens Annotation Release 110 - log base 2 scaled
RNA-seq intron features, aggregate (filtered), NCBI Homo sapiens Annotation Release 110
NC_000005.10: 133M..133M (11,296 nt)
Tracks shown: 11/951

STEP_05

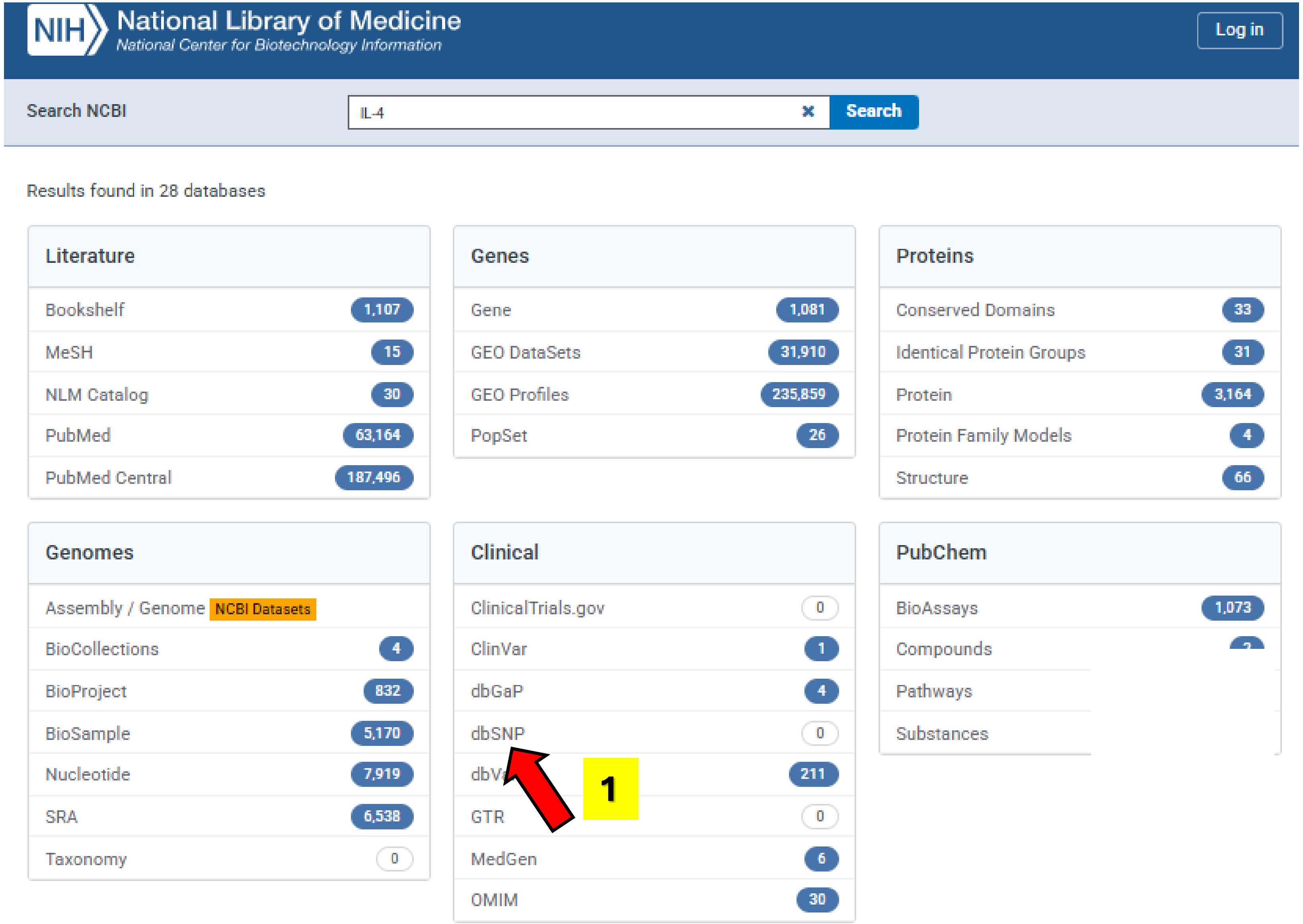
NIH
National Library of Medicine
National Center for Biotechnology Information
Log in
Search NCBI
IL-4
Search
Results found in 28 databases
Literature
Bookshelf 1,107
MeSH 15
NLM Catalog 30
PubMed 63,164
PubMed Central 187,496
Genes
Gene 1,081
GEO DataSets 31,910
GEO Profiles 235,859
PopSet 26
Proteins
Conserved Domains 33
Identical Protein Groups 31
Protein 3,164
Protein Family Models 4
Structure 66
Genomes
Assembly / Genome NCBI Datasets
BioCollections 4
BioProject 832
BioSample 5,170
Nucleotide 7,919
SRA 6,538
Taxonomy 0
Clinical
ClinicalTrials.gov 0
ClinVar 1
dbGaP 4
dbSNP 0
211
GTR 0
MedGen 6
OMIM 30
PubChem
BioAssays 1,073
Compounds
Pathways
Substances
1

STEP_05

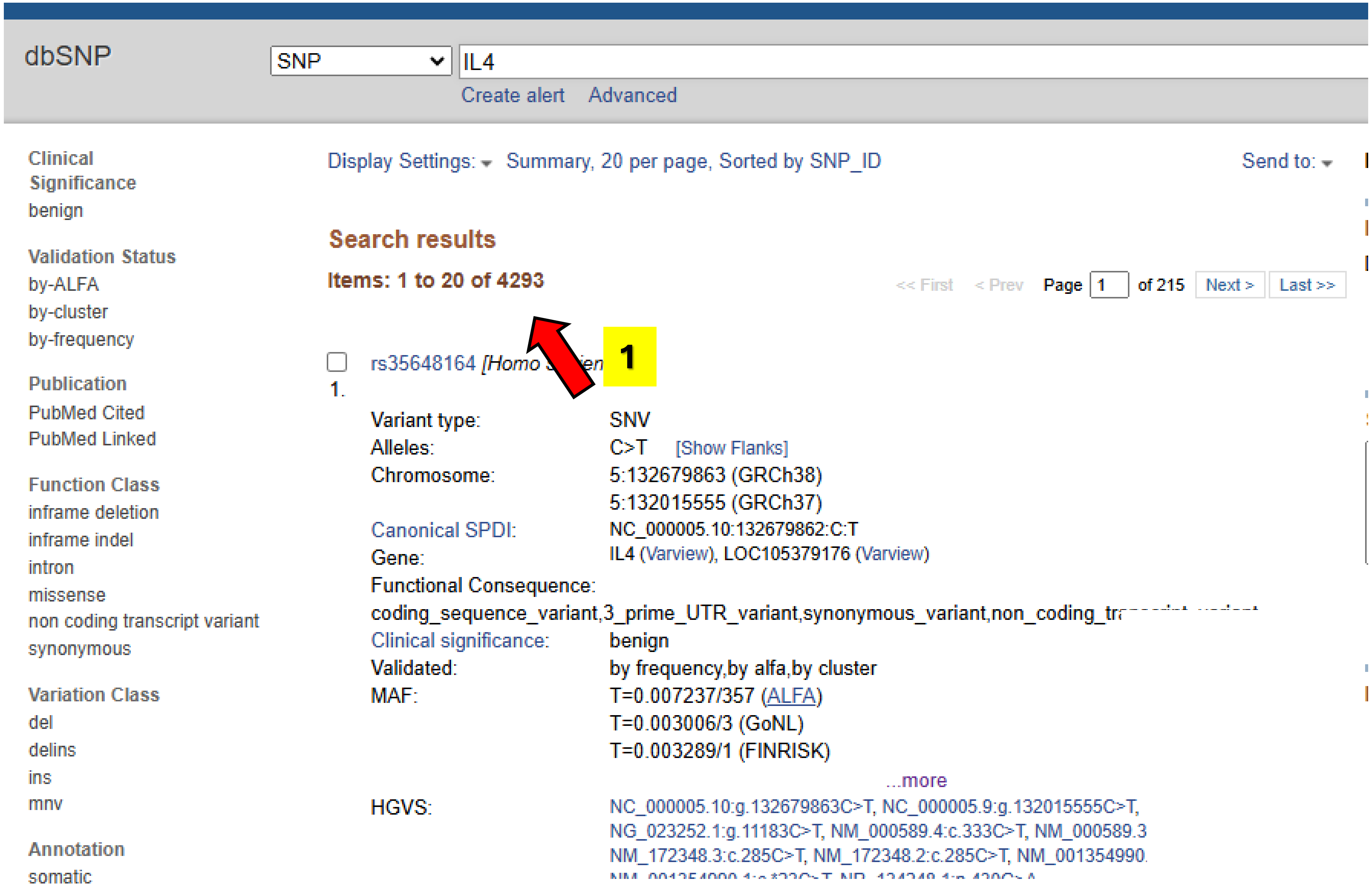
dbSNP
SNP
IL4
Create alert
Advanced
Clinical Significance
benign
Validation Status
by-ALFA
by-cluster
by-frequency
Publication
PubMed Cited
PubMed Linked
Function Class
inframe deletion
inframe indel
intron
missense
non coding transcript variant
synonymous
Variation Class
del
delins
ins
mnv
Annotation
somatic
Display Settings: Summary, 20 per page, Sorted by SNP_ID
Send to:
Search results
Items: 1 to 20 of 4293
<< First
< Prev
Page 1 of 215
Next >
Last >>
1
rs35648164 [Homo
1.
Variant type: SNV
Alleles: C>T [Show Flanks]
Chromosome: 5:132679863 (GRCh38)
5:132015555 (GRCh37)
Canonical SPDI: NC_000005.10:132679862:C:T
Gene: IL4 (Varview), LOC105379176 (Varview)
Functional Consequence:
coding_sequence_variant,3_prime_UTR_variant,synonymous_variant,non_coding_tr
Clinical significance: benign
Validated: by frequency,by alfa,by cluster
MAF: T=0.007237/357 (ALFA)
T=0.003006/3 (GoNL)
T=0.003289/1 (FINRISK)
...more
HGVS: NC_000005.10:g.132679863C>T, NC_000005.9:g.132015555C>T,
NG_023252.1:g.11183C>T, NM_000589.4:c.333C>T, NM_000589.3
NM_172348.3:c.285C>T, NM_172348.2:c.285C>T, NM_001354990.

STEP_05

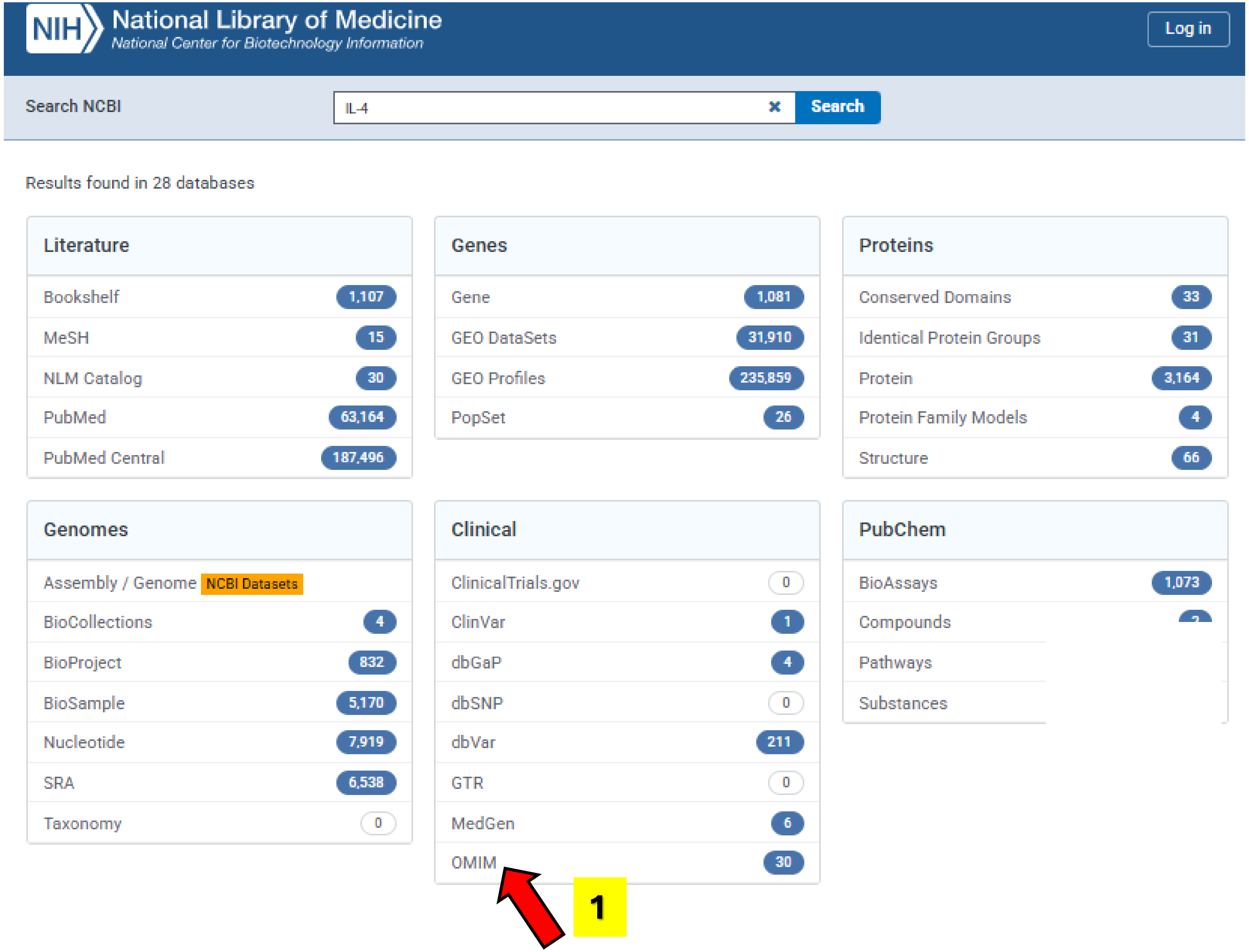
NIH
National Library of Medicine
National Center for Biotechnology Information
Log in
Search NCBI
IL-4
Search
Results found in 28 databases
Literature
Bookshelf 1,107
MeSH 15
NLM Catalog 30
PubMed 63,164
PubMed Central 187,496
Genes
Gene 1,081
GEO DataSets 31,910
GEO Profiles 235,859
PopSet 26
Proteins
Conserved Domains 33
Identical Protein Groups 31
Protein 3,164
Protein Family Models 4
Structure 66
Genomes
Assembly / Genome NCBI Datasets
BioCollections 4
BioProject 832
BioSample 5,170
Nucleotide 7,919
SRA 6,538
Taxonomy 0
Clinical
ClinicalTrials.gov 0
ClinVar 1
dbGaP 4
dbSNP 0
dbVar 211
GTR 0
MedGen 6
OMIM 30
PubChem
BioAssays 1,073
Compounds
Pathways
Substances
1

STEP_05

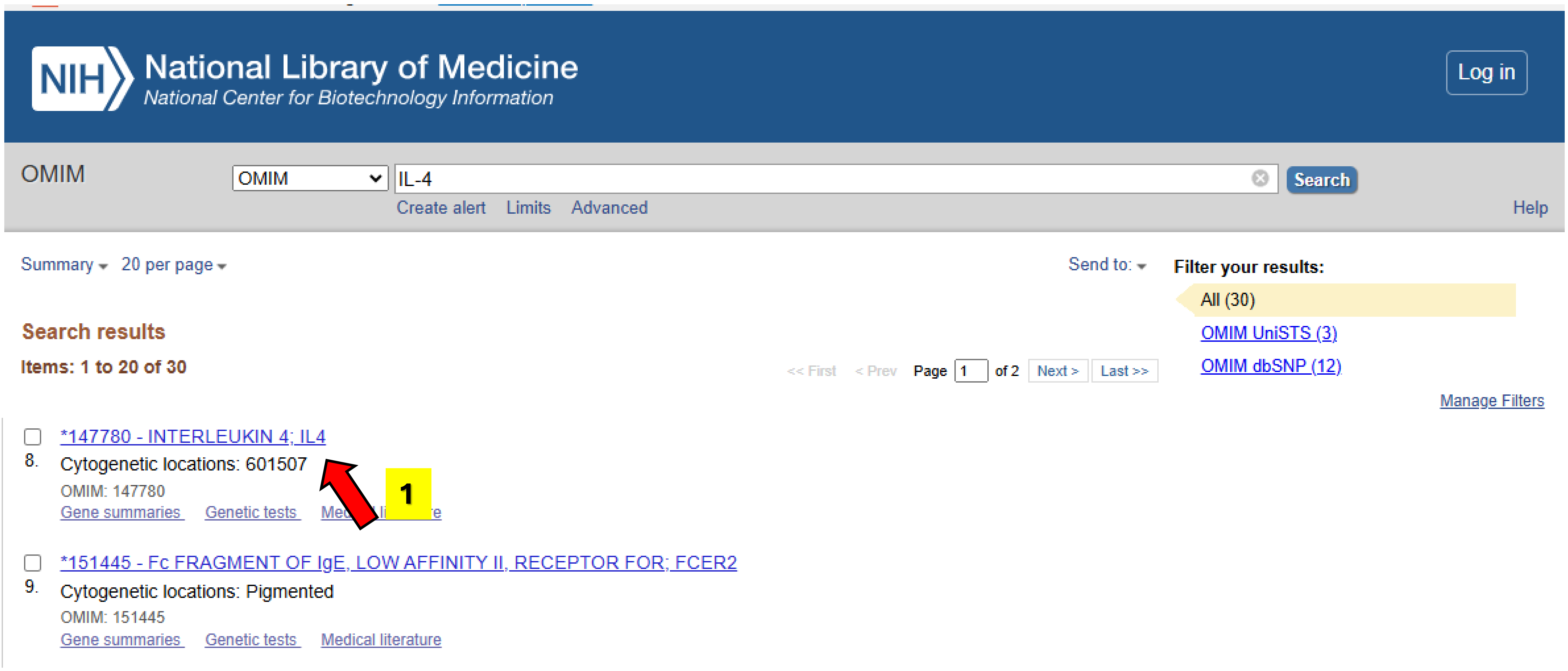

National Library of Medicine
National Center for Biotechnology Information
Log in
OMIM
OMIM
IL-4
Search
Create alert
Limits
Advanced
Help
Summary
20 per page
Send to:
Filter your results:
All (30)
OMIM UniSTS (3)
OMIM dbSNP (12)
Manage Filters
Search results
Items: 1 to 20 of 30
<< First
< Prev
Page 1 of 2
Next >
Last >>
8.
*147780 - INTERLEUKIN 4; IL4
Cytogenetic locations: 601507
OMIM: 147780
Gene summaries
Genetic tests
1
9.
*151445 - Fc FRAGMENT OF IgE, LOW AFFINITY II, RECEPTOR FOR; FCER2
Cytogenetic locations: Pigmented
OMIM: 151445
Gene summaries
Genetic tests
Medical literature

STEP_05

About | Statistics | Downloads | Contact Us | MIMmatch | Donate | Help

Search OMIM... | Options

*147780
Table of Contents

- Title
- Text
  - Cloning and Expression
  - Gene Structure
  - Mapping
  - Gene Function
  - Biochemical Features
  - Molecular Genetics
  - Animal Model
- See Also
- References
- Contributors
- Creation Date
- Edit History

* 147780

INTERLEUKIN 4; IL4

Alternative titles; symbols

B-CELL STIMULATORY FACTOR 1; BSF1

HGNC Approved Gene Symbol: *IL4*

Cytogenetic location: *5q31.1* ***Genomic coordinates*** (GRCh38) : *5:132,673,989-132,682,678* (from NCBI)

TEXT

▼ Cloning and Expression

The proliferation and differentiation of B cells is mediated in part by soluble factors produced by lectin- or antigen-activated T cells. In mice, at least 2 distinct B-cell growth factors have been described. One of these is called BSF1. Two laboratories isolated cDNA clones encoding a polypeptide with BSF1 activity from a cDNA library made with mRNA from conconavalin A-activated mouse helper T cells. Based on homology to mouse Bsf1 cDNA, Yokota et al. (1986) isolated the human equivalent from a cDNA library of conconavalin A-activated human T cells. The human cDNA contained a single open reading frame encoding a protein of 153 amino acids, including a putative signal peptide. The mouse and human genes and their protein products show structural and functional similarities. The lymphokine, which they termed IL4, also has T-cell and mast cell growth factor activities distinct from IL2 (147680) and IL3 (147740). IL4 is an 18-kD glycoprotein.

▼ External Links

- Genome
- DNA
- Protein
- Gene Info
- Clinical Resources
- Variation
- Animal Models
- Cellular Pathways

1

STEP_06

*147780
Table of Contents

- Title
- Text
  - Cloning and Expression
  - Gene Structure
  - Mapping
  - Gene Function
  - Biochemical Features
  - Molecular Genetics
  - Animal Model
- See Also
- References
- Contributors
- Creation Date
- Edit History

* 147780

INTERLEUKIN 4; IL4

Alternative titles; symbols

B-CELL STIMULATORY FACTOR 1; BSF1

HGNC Approved Gene Symbol: *IL4*

Cytogenetic location: *5q31.1* ***Genomic coordinates*** *(GRCh38)* **:** *5:132,673,989-132,682,678* (from NCBI)

TEXT

▼ Cloning and Expression

The proliferation and differentiation of B cells is mediated in part by soluble factors produced by lectin- or antigen-activated T cells. In mice, at least 2 distinct B-cell growth factors have been described. One of these is called BSF1. Two laboratories isolated cDNA clones encoding a polypeptide with BSF1 activity from a cDNA library made with mRNA from conconavalin A-activated mouse helper T cells. Based on homology to mouse Bsf1 cDNA, Yokota et al. (1986) isolated the human equivalent from a cDNA library of conconavalin A-activated human T cells. The human cDNA contained a single open reading frame encoding a protein of 153 amino acids, including a putative signal peptide. The mouse and human genes and their protein products show structural and functional similarities. The lymphokine, which they termed IL4, also has T-cell and mast cell growth factor activities distinct from IL2 (147680) and IL3 (147740). IL4 is an 18-kD glycoprotein.

▼ External Links

▼ Genome

- Ensembl
- NC[illegible]nome Viewer
- U[illegible]enom[illegible] Brow[illegible]

1

- ▶ DNA
- ▶ Protein
- ▶ Gene Info
- ▶ Clinical Resources
- ▶ Variation
- ▶ Animal Models
- ▶ Cellular Pathways

STEP_06

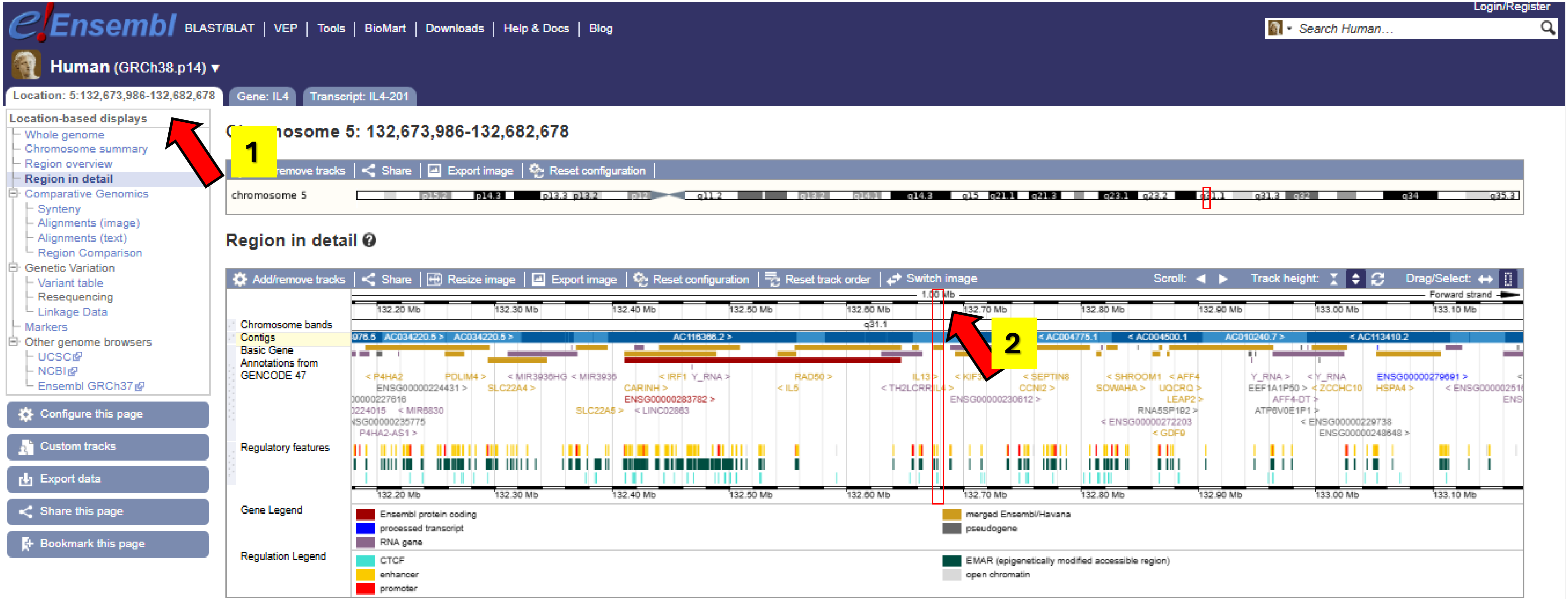
Ensembl
BLAST/BLAT | VEP | Tools | BioMart | Downloads | Help & Docs | Blog
Login/Register
Search Human…
Human (GRCh38.p14)
Location: 5:132,673,986-132,682,678
Gene: IL4
Transcript: IL4-201
Location-based displays
Whole genome
Chromosome summary
Region overview
Region in detail
Comparative Genomics
Synteny
Alignments (image)
Alignments (text)
Region Comparison
Genetic Variation
Variant table
Resequencing
Linkage Data
Markers
Other genome browsers
UCSC
NCBI
Ensembl GRCh37
Configure this page
Custom tracks
Export data
Share this page
Bookmark this page
1
2
osome 5: 132,673,986-132,682,678
emove tracks
Share
Export image
Reset configuration
chromosome 5
Region in detail
Add/remove tracks
Resize image
Reset track order
Switch image
Scroll:
Track height:
Drag/Select:
Forward strand
Chromosome bands
Contigs
Basic Gene Annotations from GENCODE 47
Regulatory features
Gene Legend
Ensembl protein coding
processed transcript
RNA gene
merged Ensembl/Havana
pseudogene
Regulation Legend
CTCF
enhancer
promoter
EMAR (epigenetically modified accessible region)
open chromatin

STEP_06

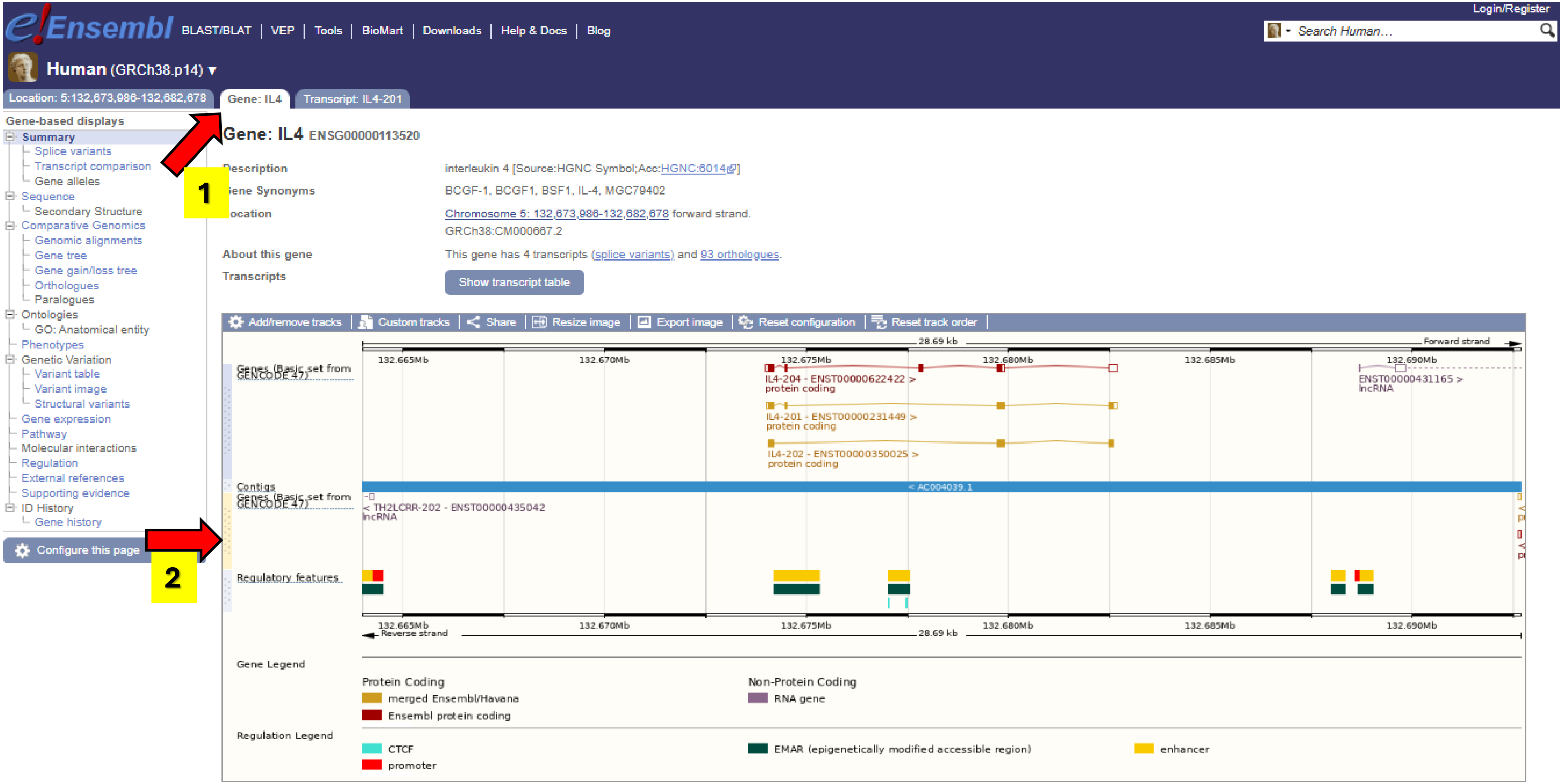

Ensembl
BLAST/BLAT | VEP | Tools | BioMart | Downloads | Help & Docs | Blog
Login/Register
Search Human…
Human (GRCh38.p14)
Location: 5:132,673,986-132,682,678
Gene: IL4
Transcript: IL4-201
Gene-based displays
Summary
Splice variants
Transcript comparison
Gene alleles
Sequence
Secondary Structure
Comparative Genomics
Genomic alignments
Gene tree
Gene gain/loss tree
Orthologues
Paralogues
Ontologies
GO: Anatomical entity
Phenotypes
Genetic Variation
Variant table
Variant image
Structural variants
Gene expression
Pathway
Molecular interactions
Regulation
External references
Supporting evidence
ID History
Gene history
Configure this page
1
2
Gene: IL4 ENSG00000113520
Description
interleukin 4 [Source:HGNC Symbol;Acc:HGNC:6014]
Gene Synonyms
BCGF-1, BCGF1, BSF1, IL-4, MGC79402
Location
Chromosome 5: 132,673,986-132,682,678 forward strand.
GRCh38:CM000667.2
About this gene
This gene has 4 transcripts (splice variants) and 93 orthologues.
Transcripts
Show transcript table
Add/remove tracks
Custom tracks
Share
Resize image
Export image
Reset configuration
Reset track order
28.69 kb
Forward strand
132.665Mb
132.670Mb
132.675Mb
132.680Mb
132.685Mb
132.690Mb
Genes (Basic set from GENCODE 47)
IL4-204 - ENST00000622422 >
protein coding
IL4-201 - ENST00000231449 >
protein coding
IL4-202 - ENST00000350025 >
protein coding
ENST00000431165 >
lncRNA
Contigs
< AC004039.1
< TH2LCRR-202 - ENST00000435042
lncRNA
Regulatory features
Reverse strand
Gene Legend
Protein Coding
merged Ensembl/Havana
Ensembl protein coding
Non-Protein Coding
RNA gene
Regulation Legend
CTCF
promoter
EMAR (epigenetically modified accessible region)
enhancer

STEP_07

About | Statistics | Downloads | Contact Us | MIMmatch | Donate | Help

Search OMIM... | Options

*147780
Table of Contents

- Title
- Text
  - Cloning and Expression
  - Gene Structure
  - Mapping
  - Gene Function
  - Biochemical Features
  - Molecular Genetics
  - Animal Model
- See Also
- References
- Contributors
- Creation Date
- Edit History

* 147780

INTERLEUKIN 4; IL4

Alternative titles; symbols

B-CELL STIMULATORY FACTOR 1; BSF1

HGNC Approved Gene Symbol: IL4

Cytogenetic location: 5q31.1 ***Genomic coordinates*** (GRCh38) : 5:132,673,989-132,682,678 (from NCBI)

TEXT

Cloning and Expression

The proliferation and differentiation of B cells is mediated in part by soluble factors produced by lectin- or antigen-activated T cells. In mice, at least 2 distinct B-cell growth factors have been described. One of these is called BSF1. Two laboratories isolated cDNA clones encoding a polypeptide with BSF1 activity from a cDNA library made with mRNA from conconavalin A-activated mouse helper T cells. Based on homology to mouse Bsf1 cDNA, Yokota et al. (1986) isolated the human equivalent from a cDNA library of conconavalin A-activated human T cells. The human cDNA contained a single open reading frame encoding a protein of 153 amino acids, including a putative signal peptide. The mouse and human genes and their protein products show structural and functional similarities. The lymphokine, which they termed IL4, also has T-cell and mast cell growth factor activities distinct from IL2 (147680) and IL3 (147740). IL4 is an 18-kD glycoprotein.

External Links

- Genome
- DNA
  - Ensembl (MANE Select)
  - NCBI RefS[illegible]
  - NCBI RefSeq [illegible]NE Select)
  - UCSC Genome Browser
- Protein
- Gene Info
- Clinical Resources
- Variation
- Animal Models
- Cellular Pathways

1

STEP_07

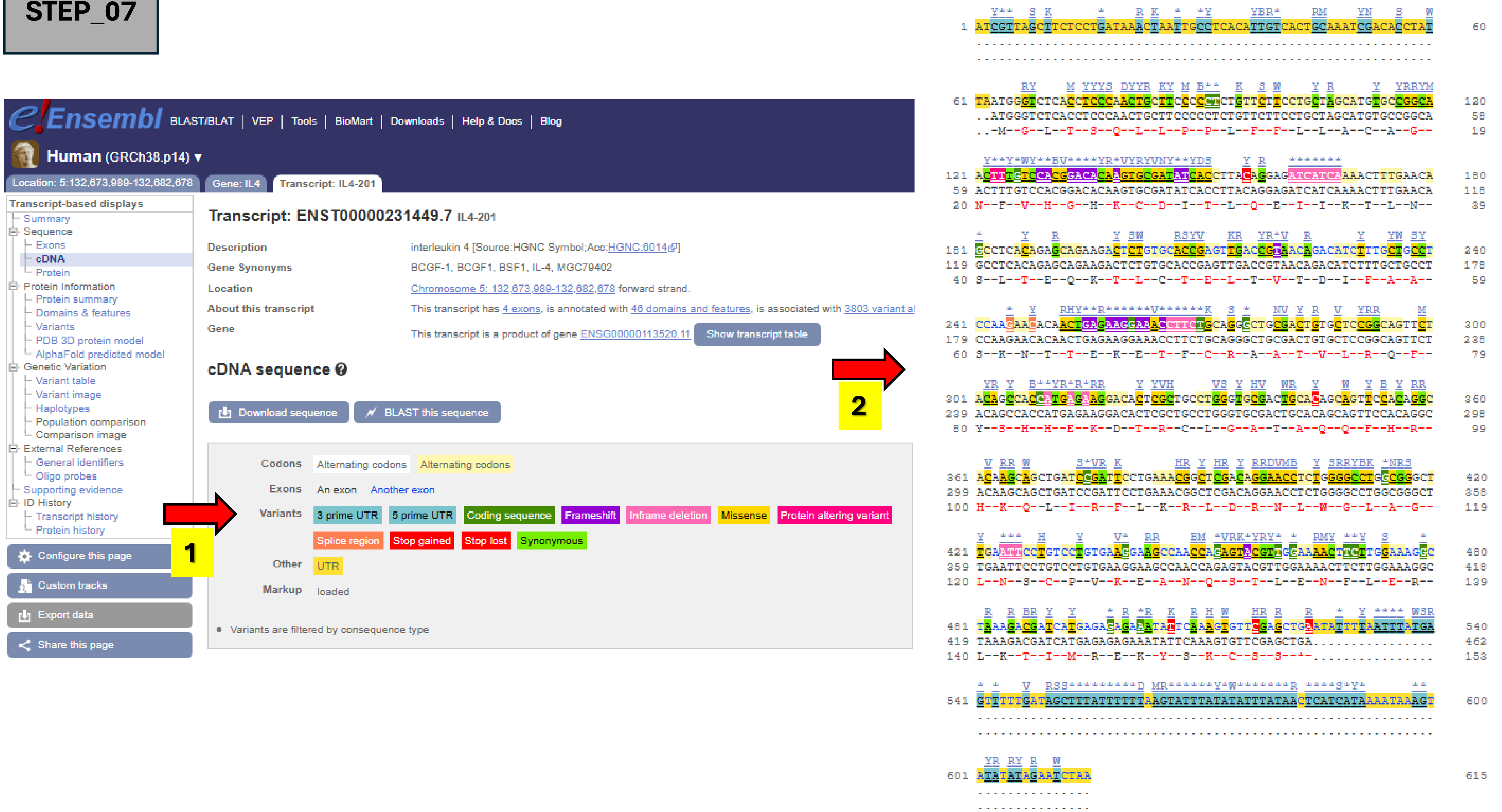

Ensembl
BLAST/BLAT | VEP | Tools | BioMart | Downloads | Help & Docs | Blog
Human (GRCh38.p14)
Location: 5:132,673,989-132,682,678
Gene: IL4
Transcript: IL4-201
Transcript-based displays
Summary
Sequence
Exons
cDNA
Protein
Protein Information
Protein summary
Domains & features
Variants
PDB 3D protein model
AlphaFold predicted model
Genetic Variation
Variant table
Variant image
Haplotypes
Population comparison
Comparison image
External References
General identifiers
Oligo probes
Supporting evidence
ID History
Transcript history
Protein history
Configure this page
Custom tracks
Export data
Share this page
Transcript: ENST00000231449.7 IL4-201
Description
interleukin 4 [Source:HGNC Symbol;Acc:HGNC:6014]
Gene Synonyms
BCGF-1, BCGF1, BSF1, IL-4, MGC79402
Location
Chromosome 5: 132,673,989-132,682,678 forward strand.
About this transcript
This transcript has 4 exons, is annotated with 46 domains and features, is associated with 3803 variant al
Gene
This transcript is a product of gene ENSG00000113520.11
Show transcript table
cDNA sequence
Download sequence
BLAST this sequence
Codons
Alternating codons
Alternating codons
Exons
An exon
Another exon
Variants
3 prime UTR
5 prime UTR
Coding sequence
Frameshift
Inframe deletion
Missense
Protein altering variant
Splice region
Stop gained
Stop lost
Synonymous
Other
UTR
Markup
loaded
Variants are filtered by consequence type
1
2

STEP_08

About | Statistics | Downloads | Contact Us | MIMmatch | Donate | Help

Search OMIM... | Options

*147780
Table of Contents

- Title
- Text
  - Cloning and Expression
  - Gene Structure
  - Mapping
  - Gene Function
  - Biochemical Features
  - Molecular Genetics
  - Animal Model
- See Also
- References
- Contributors
- Creation Date
- Edit History

* 147780

INTERLEUKIN 4; IL4

Alternative titles; symbols

B-CELL STIMULATORY FACTOR 1; BSF1

HGNC Approved Gene Symbol: *IL4*

Cytogenetic location: *5q31.1* ***Genomic coordinates (GRCh38) :*** *5:132,673,989-132,682,678* (from NCBI)

TEXT

▼ Cloning and Expression

The proliferation and differentiation of B cells is mediated in part by soluble factors produced by lectin- or antigen-activated T cells. In mice, at least 2 distinct B-cell growth factors have been described. One of these is called BSF1. Two laboratories isolated cDNA clones encoding a polypeptide with BSF1 activity from a cDNA library made with mRNA from conconavalin A-activated mouse helper T cells. Based on homology to mouse Bsf1 cDNA, Yokota et al. (1986) isolated the human equivalent from a cDNA library of conconavalin A-activated human T cells. The human cDNA contained a single open reading frame encoding a protein of 153 amino acids, including a putative signal peptide. The mouse and human genes and their protein products show structural and functional similarities. The lymphokine, which they termed IL4, also has T-cell and mast cell growth factor activities distinct from IL2 (147680) and IL3 (147740). IL4 is an 18-kD glycoprotein.

▼ External Links

- ► Genome
- ► DNA
- ▼ Protein
  - HPRD
  - Human Protein Atlas
  - NCBI Protein
  - UniProt
- ► Gene Info
- ► Clinical Resources
- ► Variation
- ► Animal Models
- ► Cellular Pathways

1

Ativar o Windc
Acesse Configuraçõ

STEP_08

Function

Cytokine secreted [illegible] by mast cells, T-cells, eosinophils, and basophils that plays a role in regulating antibody production, hematopoiesis and inflammation, and the development of effector T-cell responses (PubM[illegible]93171[illegible]d:3016727).

1

Induces the expressi[illegible] clas[illegible]C molecules on resting B-cells. Enhances both secretion and cell surface expression of IgE and IgG1 (PubMed:1993171).

Regulates also the expr[illegible]sion of the low affinity Fc receptor for IgE (CD23) on both lymphocytes and monocytes (PubMed:2521231).

Positively regulates IL31RA expression in macrophages. Stimulates autophagy in dendritic cells by interfering with mTORC1 signaling and through the induction of RUFY4. In addition, plays a critical role in higher functions of the normal brain, such as memory and learning (By similarity).

Upon binding to IL4, IL4R receptor dimerizes either with the common IL2R gamma chain/IL2RG to produce the type 1 signaling complex, located mainly on hematopoietic cells, or with the IL13RA1 to produce the type 2 complex, which is expressed also on nonhematopoietic cells (PubMed:10219247, PubMed:11526337, PubMed:18243101).

Engagement of both types of receptors initiates JAK3 and to a lower extend JAK1 phosphorylation leading to activation of the signal transducer and activator of transcription 6/S[illegible] (PubMed:7721895). By Similarity 3 Publications

GO annotations

Access the complete set of GO annotations on QuickGO

Slimming set:

generic

2

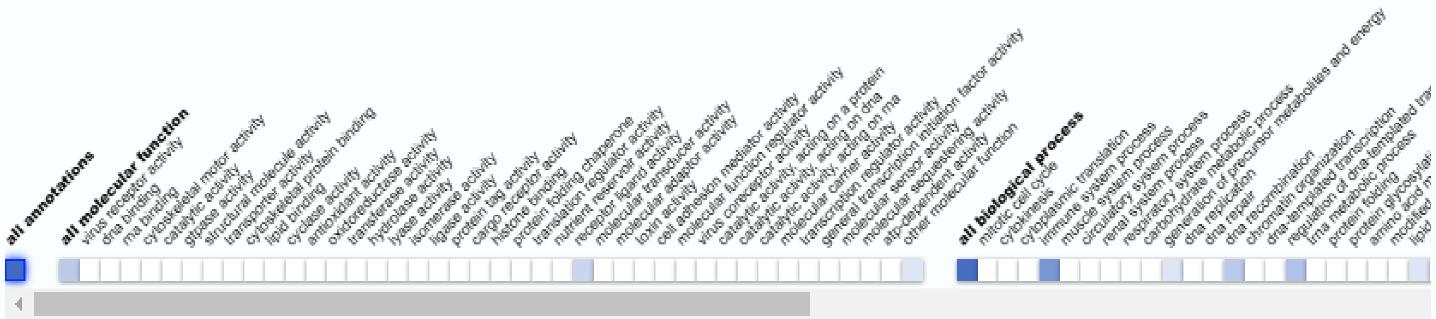

Cell color indicative of number of GO terms

Subcellular Location

UniProt Annotation GO Annotation

3

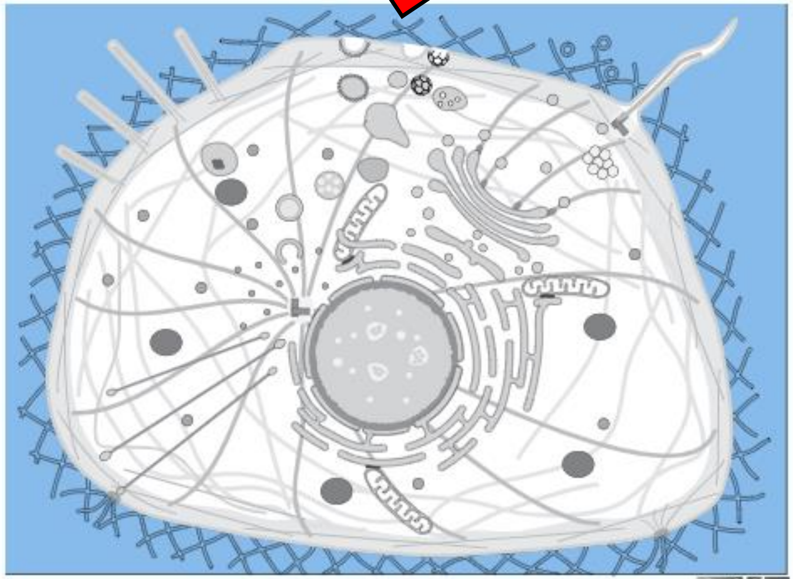

Secreted

Keywords

STEP_09

About | Statistics | Downloads | Contact Us | MIMmatch | Donate | Help

Search OMIM... | Options

*147780
Table of Contents

- Title
- Text
  - Cloning and Expression
  - Gene Structure
  - Mapping
  - Gene Function
  - Biochemical Features
  - Molecular Genetics
  - Animal Model
- See Also
- References
- Contributors
- Creation Date
- Edit History

* 147780

INTERLEUKIN 4; IL4

Alternative titles; symbols

B-CELL STIMULATORY FACTOR 1; BSF1

HGNC Approved Gene Symbol: IL4

Cytogenetic location: 5q31.1 ***Genomic coordinates (GRCh38) : 5:132,673,989-132,682,678*** (from NCBI)

TEXT

Cloning and Expression

The proliferation and differentiation of B cells is mediated in part by soluble factors produced by lectin- or antigen-activated T cells. In mice, at least 2 distinct B-cell growth factors have been described. One of these is called BSF1. Two laboratories isolated cDNA clones encoding a polypeptide with BSF1 activity from a cDNA library made with mRNA from conconavalin A-activated mouse helper T cells. Based on homology to mouse Bsf1 cDNA, Yokota et al. (1986) isolated the human equivalent from a cDNA library of conconavalin A-activated human T cells. The human cDNA contained a single open reading frame encoding a protein of 153 amino acids, including a putative signal peptide. The mouse and human genes and their protein products show structural and functional similarities. The lymphokine, which they termed IL4, also has T-cell and mast cell growth factor activities distinct from IL2 (147680) and IL3 (147740). IL4 is an 18-kD glycoprotein.

External Links

- Genome
- DNA
- Protein
- Gene Info
  - BioGPS
  - Ensembl
  - GeneCards
  - Ge[illegible]ntology
  - K[illegible]
  - MAR[illegible]
  - NCBI G[illegible]
  - UCSC
- Clinical Resources
- Variation
- Animal Models
- Cellular Pathways

1

STEP_09

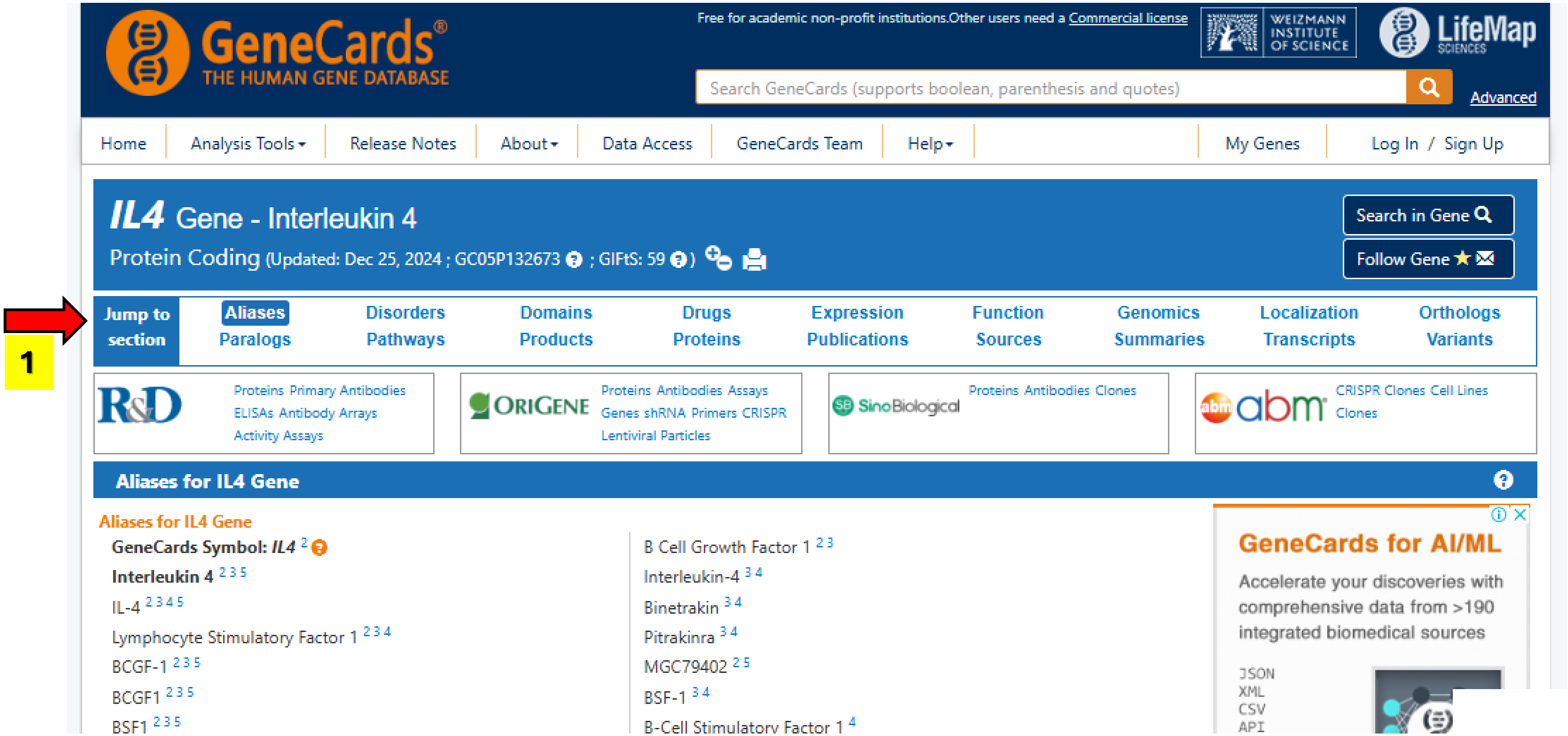
GeneCards®
THE HUMAN GENE DATABASE
Free for academic non-profit institutions.Other users need a Commercial license
WEIZMANN INSTITUTE OF SCIENCE
LifeMap SCIENCES
Search GeneCards (supports boolean, parenthesis and quotes)
Advanced
Home
Analysis Tools
Release Notes
About
Data Access
GeneCards Team
Help
My Genes
Log In / Sign Up
IL4 Gene - Interleukin 4
Protein Coding (Updated: Dec 25, 2024 ; GC05P132673 ; GIFtS: 59)
Search in Gene
Follow Gene
Jump to section
Aliases
Paralogs
Disorders
Pathways
Domains
Products
Drugs
Proteins
Expression
Publications
Function
Sources
Genomics
Summaries
Localization
Transcripts
Orthologs
Variants
1
R&D
Proteins Primary Antibodies
ELISAs Antibody Arrays
Activity Assays
ORIGENE
Proteins Antibodies Assays
Genes shRNA Primers CRISPR
Lentiviral Particles
SinoBiological
Proteins Antibodies Clones
abm
CRISPR Clones Cell Lines
Clones
Aliases for IL4 Gene
Aliases for IL4 Gene
GeneCards Symbol: IL4 2
Interleukin 4 2 3 5
IL-4 2 3 4 5
Lymphocyte Stimulatory Factor 1 2 3 4
BCGF-1 2 3 5
BCGF1 2 3 5
BSF1 2 3 5
B Cell Growth Factor 1 2 3
Interleukin-4 3 4
Binetrakin 3 4
Pitrakinra 3 4
MGC79402 2 5
BSF-1 3 4
B-Cell Stimulatory Factor 1 4
GeneCards for AI/ML
Accelerate your discoveries with comprehensive data from >190 integrated biomedical sources
JSON
XML
CSV
API

STEP_09

Localization for IL4 Gene

Subsections: Cellular C[illegible]nents

1

Subcellular locations from [illegible]rot[illegible]s-Prot for IL4 Gene

Protein: *IL4_HUMAN*

› Secreted

Subcellular locations from COMPARTMENTS

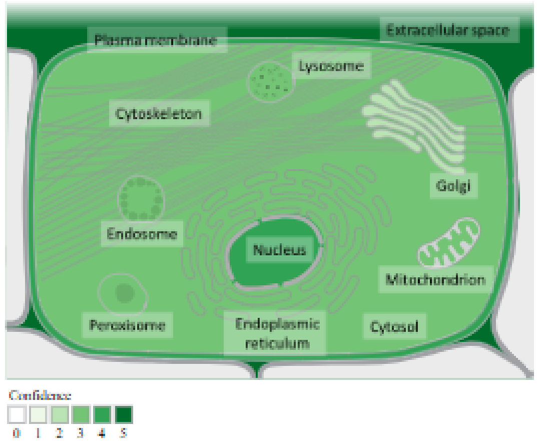

Compartment	Confidence
extracellular	5
nucleus	4
plasma membrane	4
lysosome	3
cytosol	3
endosome	3
endoplasmic reticulum	3
peroxisome	3
mitochondrion	3
cytoskeleton	3
golgi apparatus	2

Pathways & Interactions for IL4 Gene

2

Interacting Proteins for IL4 Gene

STRING Interaction Network Preview (showing top 5 STRING interacta[illegible]as[illegible]xperimental score) - click image to see top 25)

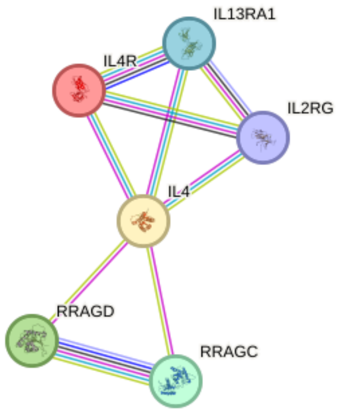

Expression for IL4 Gene

Subsections: Expression in [illegible] / Protein expression / Co-Expression partners

Buy Research Products: [illegible]

3

mRNA expression in normal human [illegible] from GTEx, Illumina, BioGPS, and SAGE for IL4 Gene

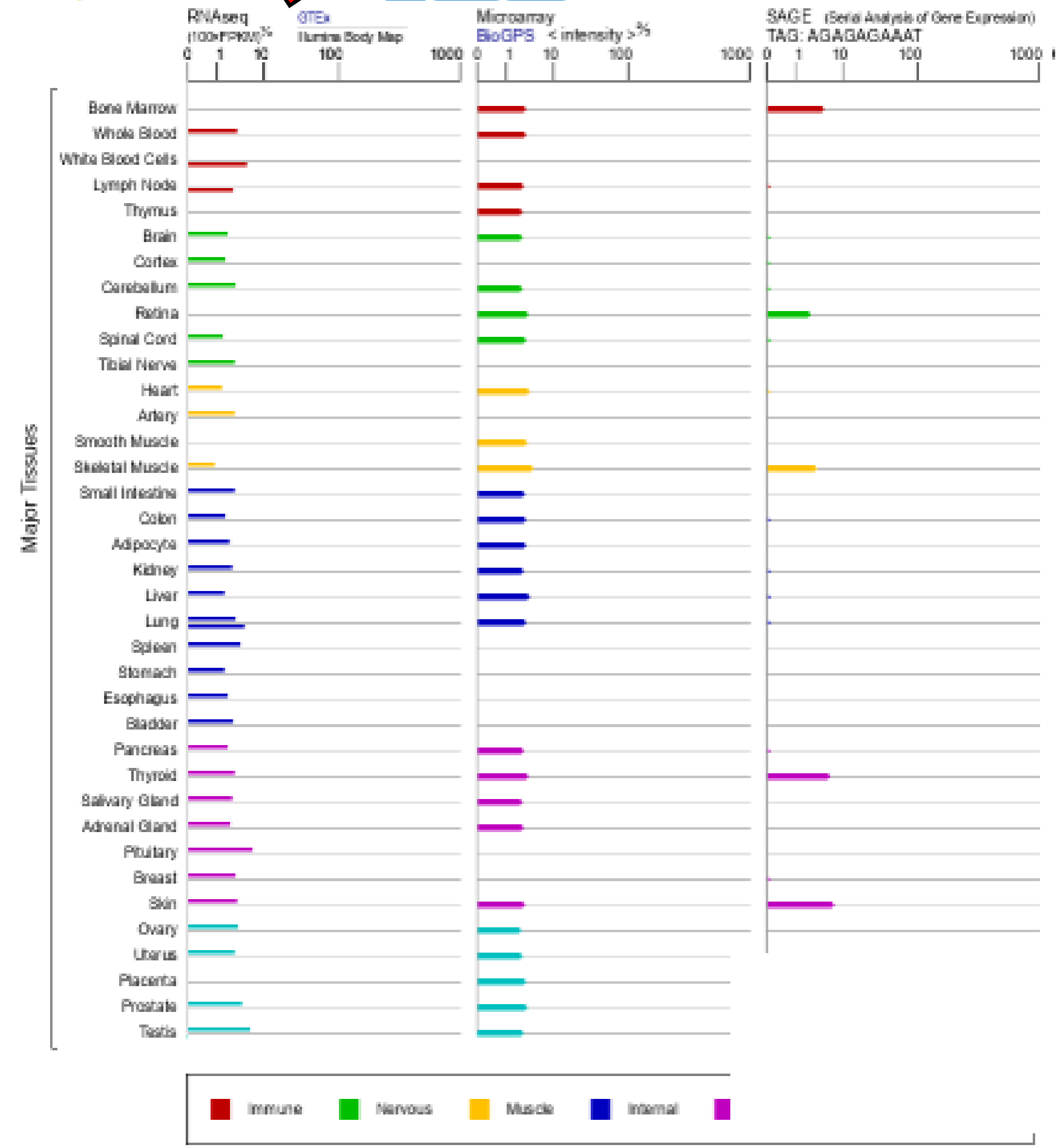

STEP_10

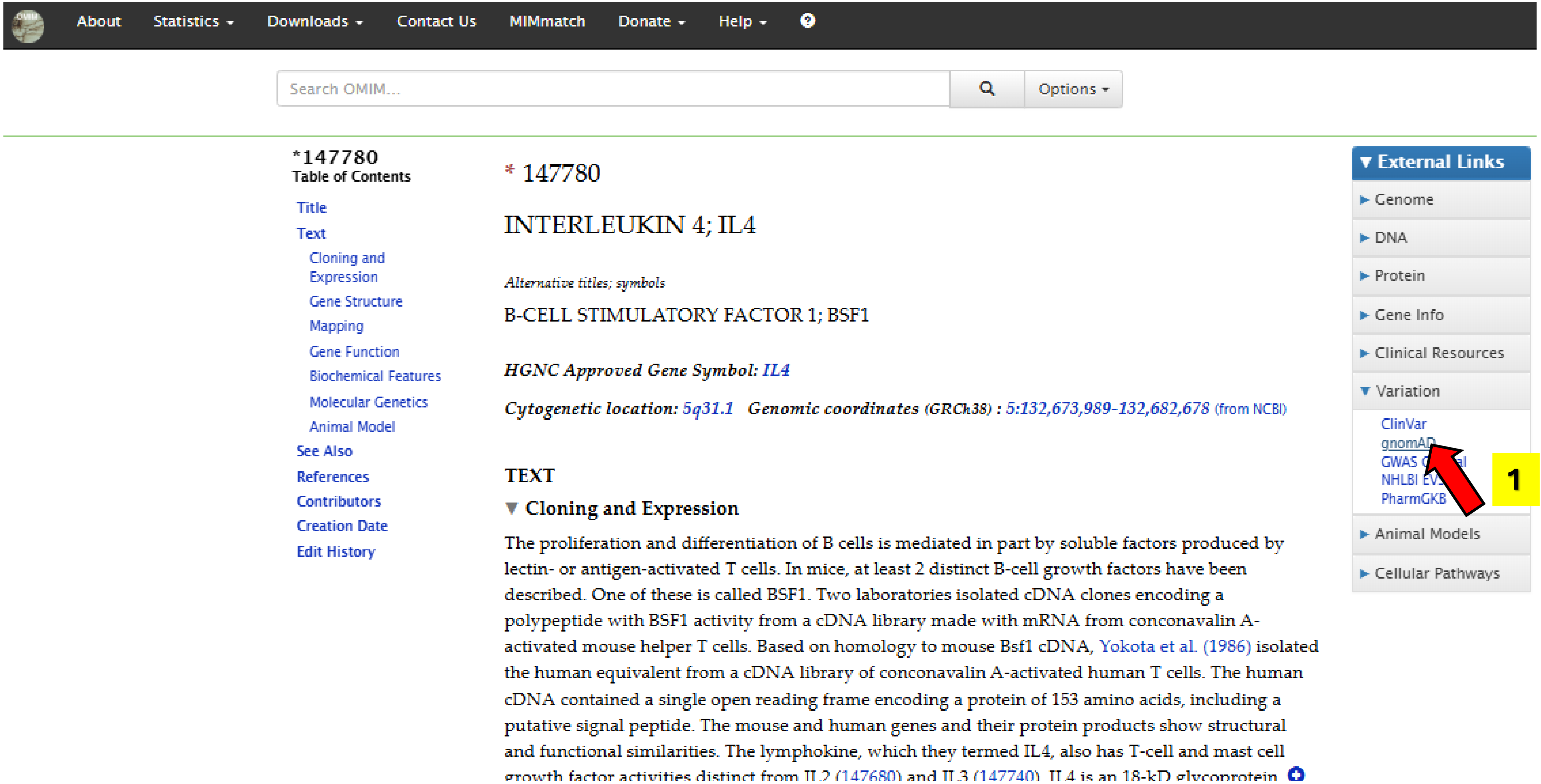

About
Statistics
Downloads
Contact Us
MIMmatch
Donate
Help
Search OMIM...
Options
*147780
Table of Contents
Title
Text
Cloning and Expression
Gene Structure
Mapping
Gene Function
Biochemical Features
Molecular Genetics
Animal Model
See Also
References
Contributors
Creation Date
Edit History
* 147780
INTERLEUKIN 4; IL4
Alternative titles; symbols
B-CELL STIMULATORY FACTOR 1; BSF1
HGNC Approved Gene Symbol: IL4
Cytogenetic location: 5q31.1 Genomic coordinates (GRCh38) : 5:132,673,989-132,682,678 (from NCBI)
TEXT
Cloning and Expression
The proliferation and differentiation of B cells is mediated in part by soluble factors produced by lectin- or antigen-activated T cells. In mice, at least 2 distinct B-cell growth factors have been described. One of these is called BSF1. Two laboratories isolated cDNA clones encoding a polypeptide with BSF1 activity from a cDNA library made with mRNA from conconavalin A-activated mouse helper T cells. Based on homology to mouse Bsf1 cDNA, Yokota et al. (1986) isolated the human equivalent from a cDNA library of conconavalin A-activated human T cells. The human cDNA contained a single open reading frame encoding a protein of 153 amino acids, including a putative signal peptide. The mouse and human genes and their protein products show structural and functional similarities. The lymphokine, which they termed IL4, also has T-cell and mast cell growth factor activities distinct from IL2 (147680) and IL3 (147740). IL4 is an 18-kD glycoprotein
External Links
Genome
DNA
Protein
Gene Info
Clinical Resources
Variation
ClinVar
gnomAD
PharmGKB
Animal Models
Cellular Pathways
1

STEP_10

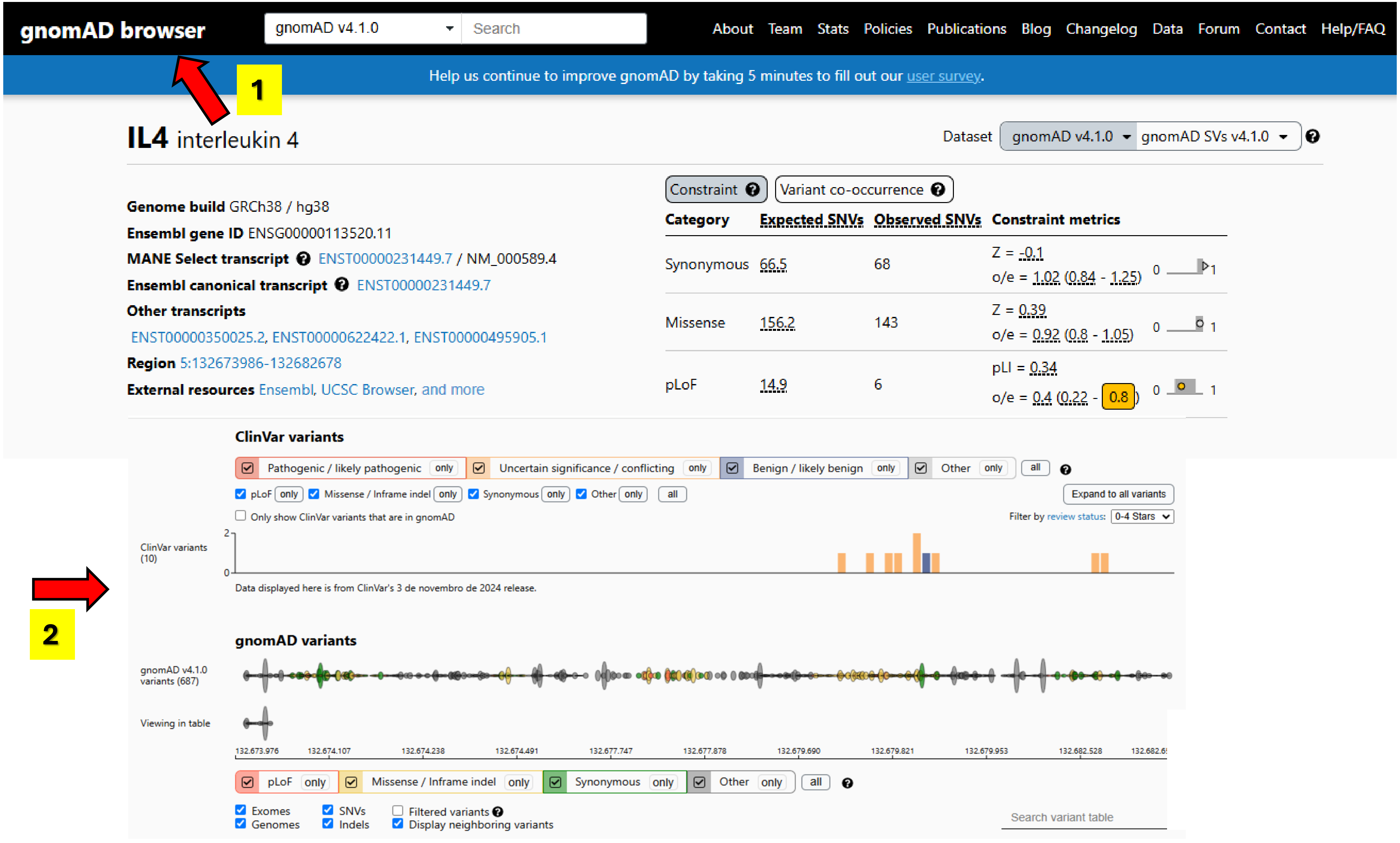

gnomAD browser
gnomAD v4.1.0
Search
About
Team
Stats
Policies
Publications
Blog
Changelog
Data
Forum
Contact
Help/FAQ
Help us continue to improve gnomAD by taking 5 minutes to fill out our user survey.
1
IL4 interleukin 4
Dataset gnomAD v4.1.0 gnomAD SVs v4.1.0
Genome build GRCh38 / hg38
Ensembl gene ID ENSG00000113520.11
MANE Select transcript ENST00000231449.7 / NM_000589.4
Ensembl canonical transcript ENST00000231449.7
Other transcripts
ENST00000350025.2, ENST00000622422.1, ENST00000495905.1
Region 5:132673986-132682678
External resources Ensembl, UCSC Browser, and more
Constraint
Variant co-occurrence
Category
Expected SNVs
Observed SNVs
Constraint metrics
Synonymous 66.5 68 Z = -0.1 o/e = 1.02 (0.84 - 1.25)
Missense 156.2 143 Z = 0.39 o/e = 0.92 (0.8 - 1.05)
pLoF 14.9 6 pLI = 0.34 o/e = 0.4 (0.22 - 0.8)
ClinVar variants
Pathogenic / likely pathogenic only
Uncertain significance / conflicting only
Benign / likely benign only
Other only
all
pLoF only
Missense / Inframe indel only
Synonymous only
Other only
all
Expand to all variants
Only show ClinVar variants that are in gnomAD
Filter by review status: 0-4 Stars
ClinVar variants (10)
Data displayed here is from ClinVar's 3 de novembro de 2024 release.
2
gnomAD variants
gnomAD v4.1.0 variants (687)
Viewing in table
pLoF only
Missense / Inframe indel only
Synonymous only
Other only
all
Exomes
Genomes
SNVs
Indels
Filtered variants
Display neighboring variants
Search variant table
