# Supplementary material for: Interactive Bioinformatics Lab: Using Genomic Databases for Active Learning in Dentistry
Source: J Dent Educ. 2025 Apr 25;89(Suppl 3):1854–8. doi: 10.1002/jdd.13911 (PMC12728793; doi:10.1002/jdd.13911)
Supplement: Supplementary file 2 — Supporting Information [file JDD-89-1854-s002.pptx]

## Slide 1
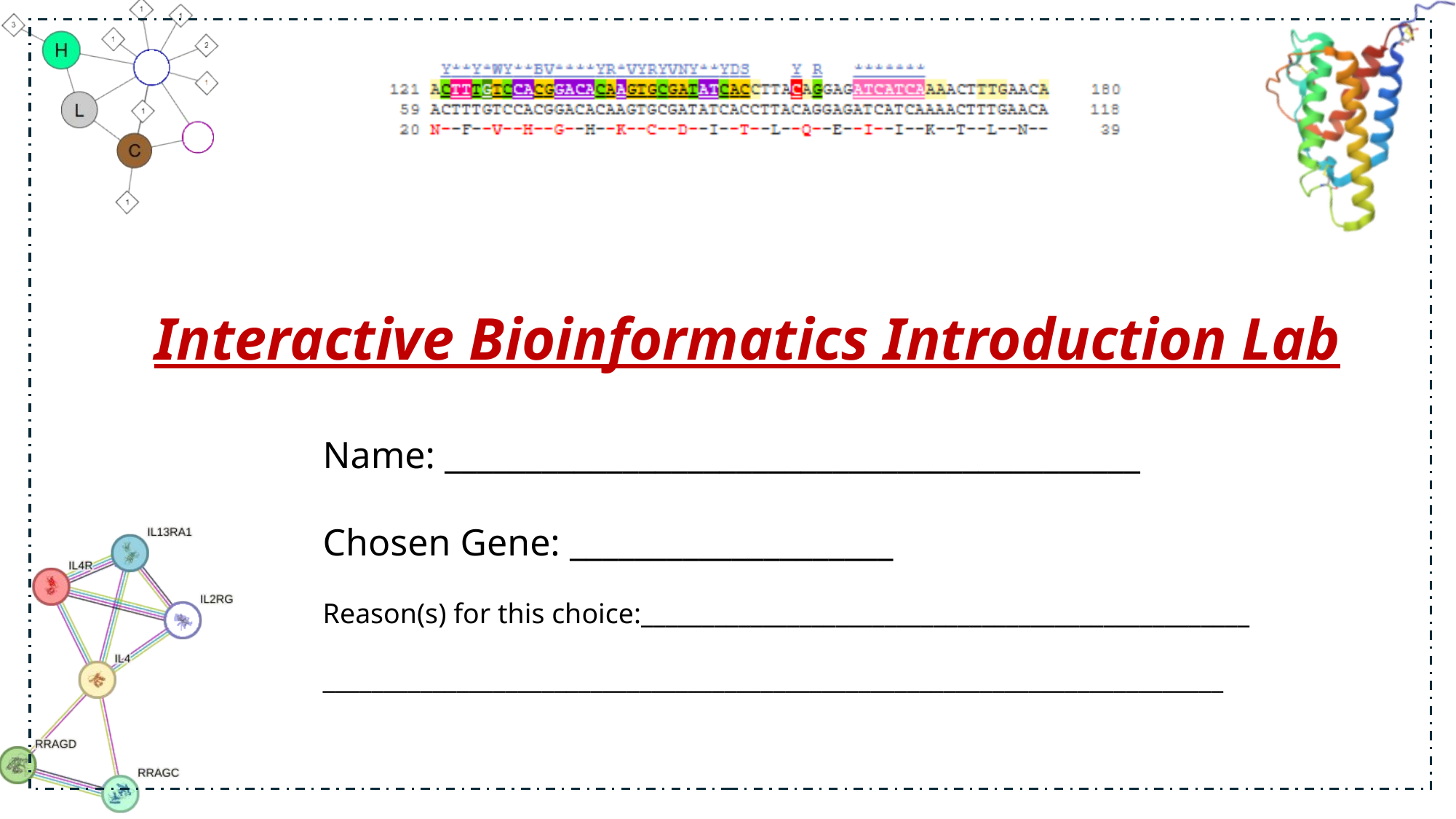

Interactive Bioinformatics Introduction Lab
Name: ___________________________________________
Chosen Gene: ____________________
Reason(s) for this choice:__________________________________________________
__________________________________________________________________________

## Slide 2
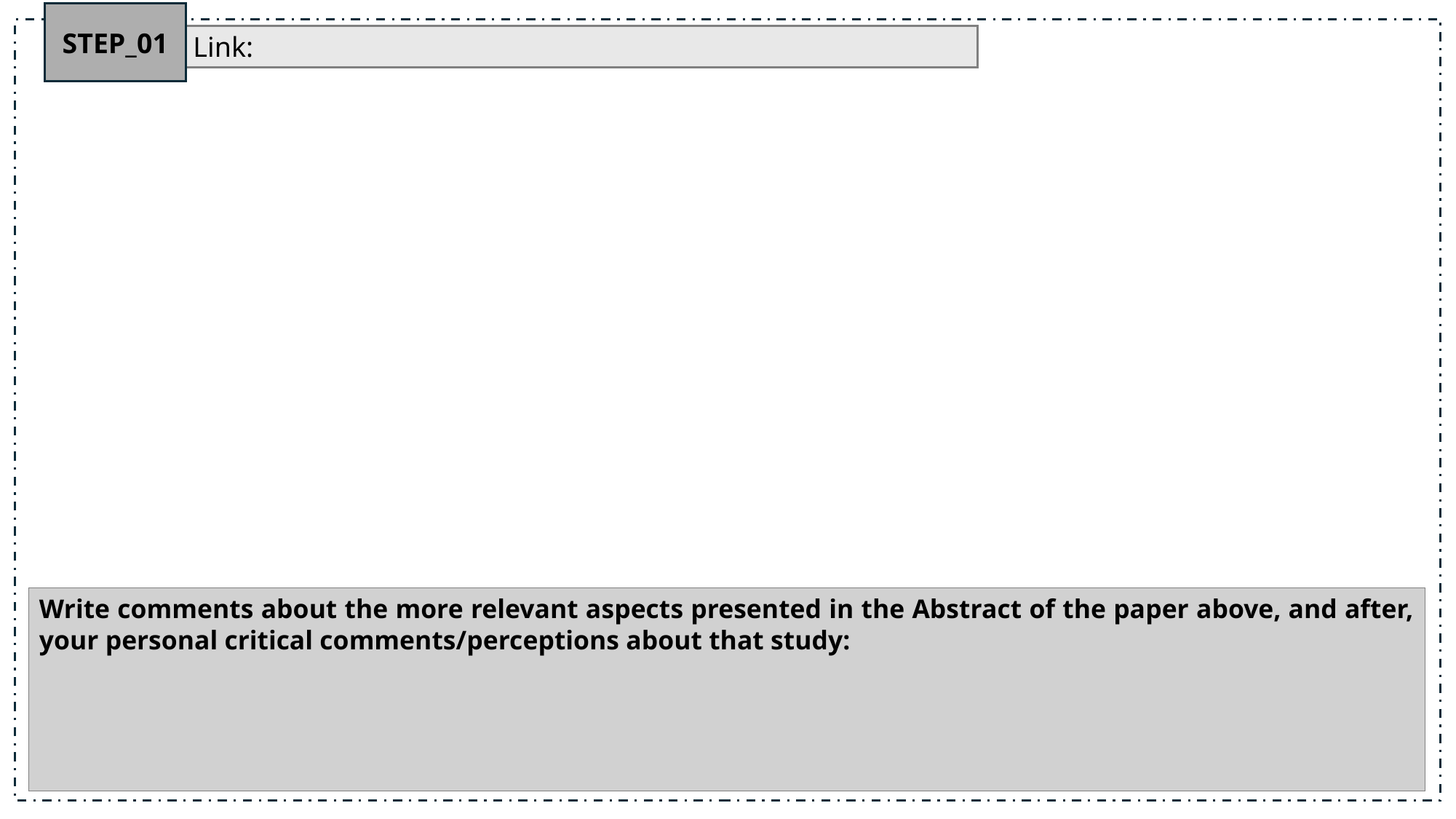

STEP_01
Link:
Write comments about the more relevant aspects presented in the Abstract of the paper above, and after, your personal critical comments/perceptions about that study:

## Slide 3
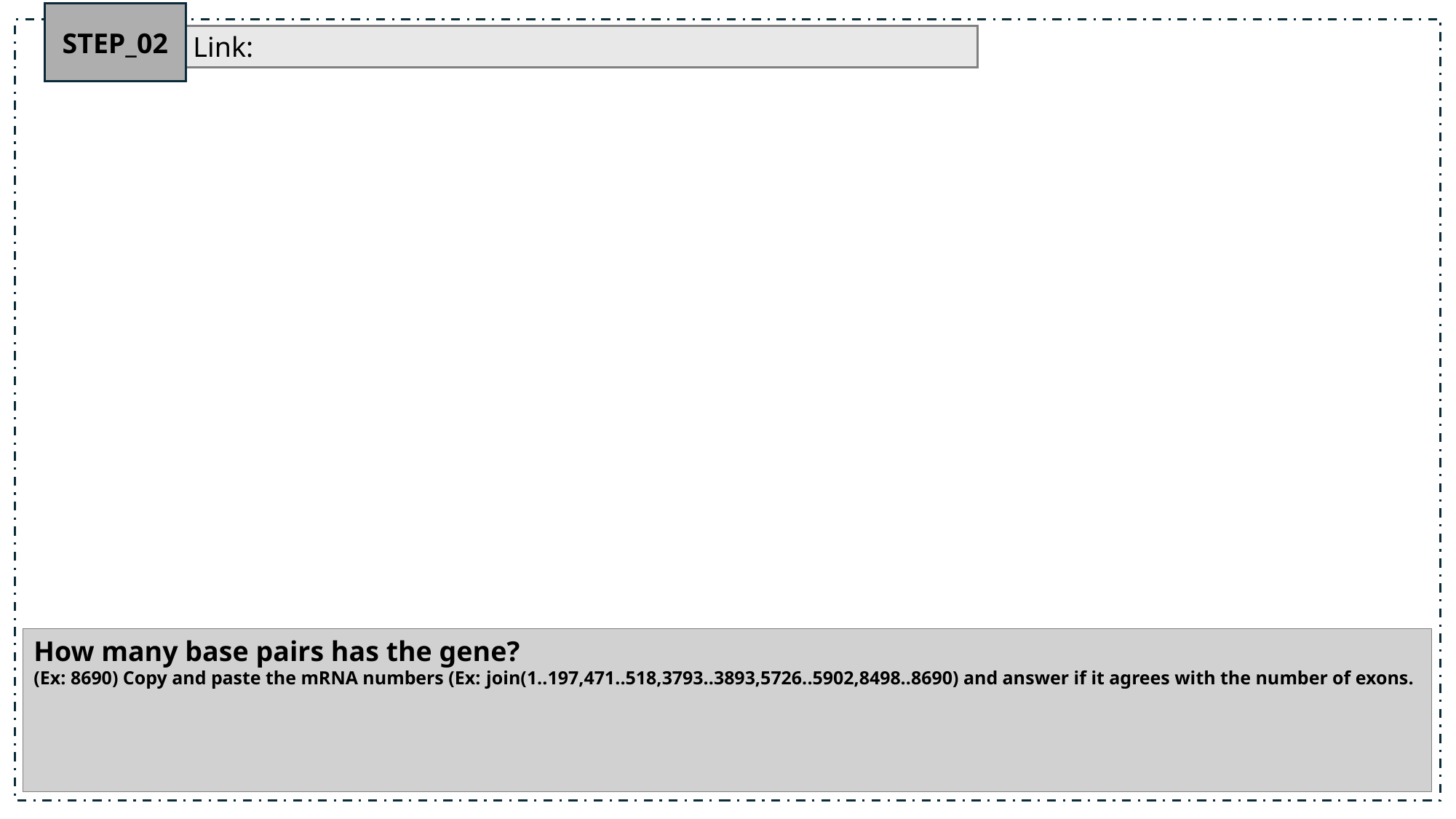

STEP_02
Link:
How many base pairs has the gene?
(Ex: 8690) Copy and paste the mRNA numbers (Ex: join(1..197,471..518,3793..3893,5726..5902,8498..8690) and answer if it agrees with the number of exons.

## Slide 4
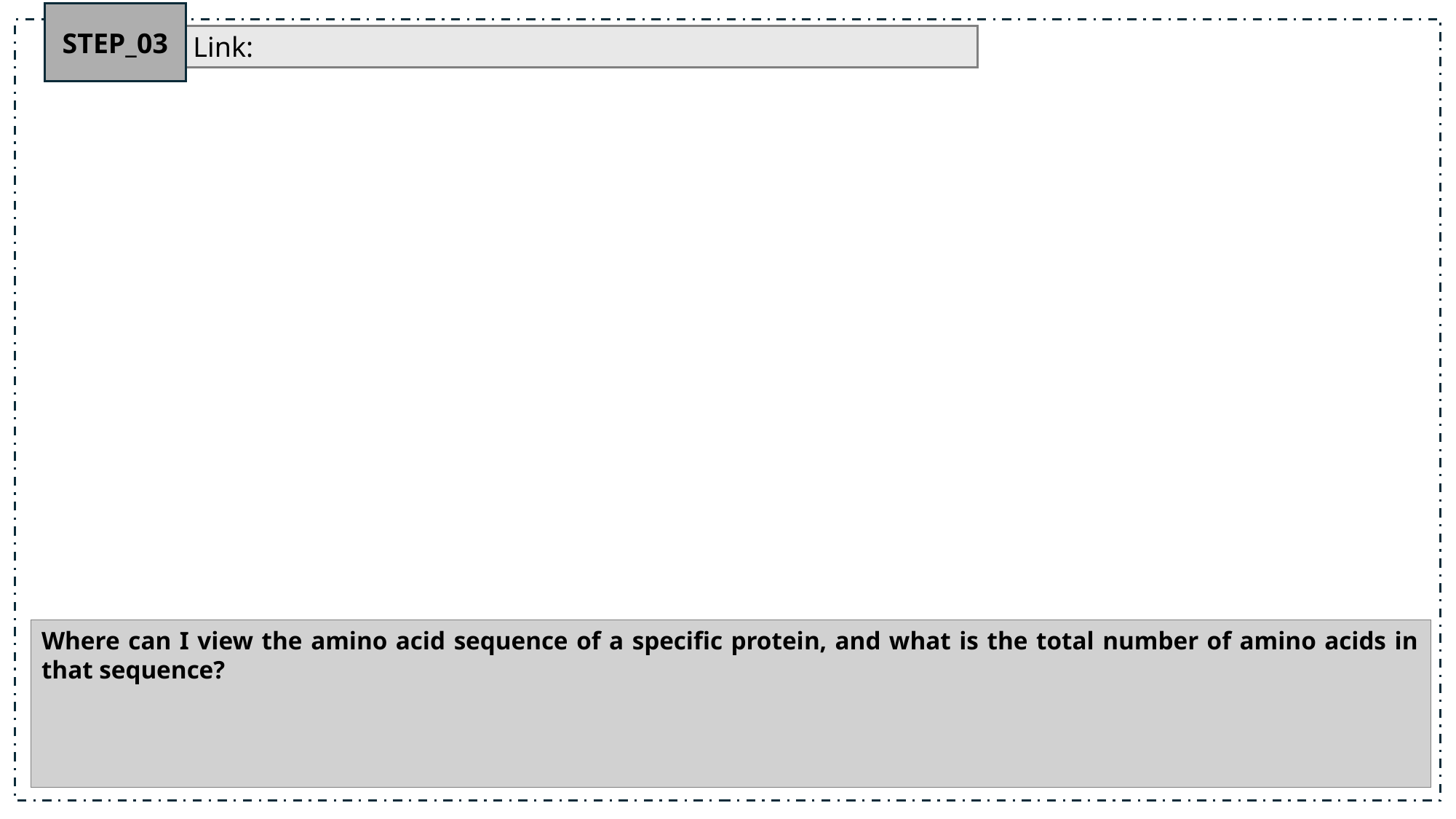

STEP_03
Link:
Where can I view the amino acid sequence of a specific protein, and what is the total number of amino acids in that sequence?

## Slide 5
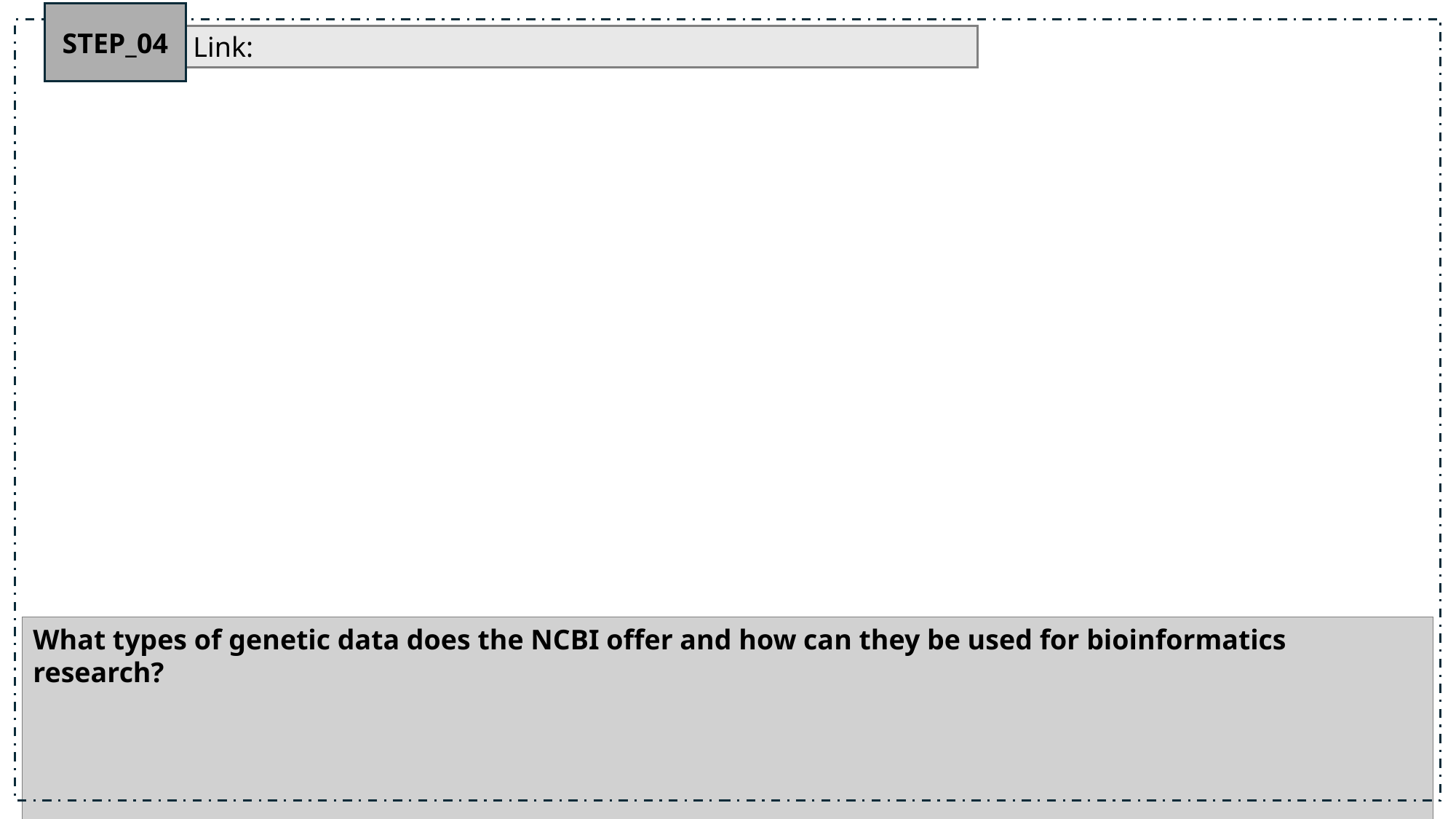

STEP_04
Link:
What types of genetic data does the NCBI offer and how can they be used for bioinformatics research?

## Slide 6
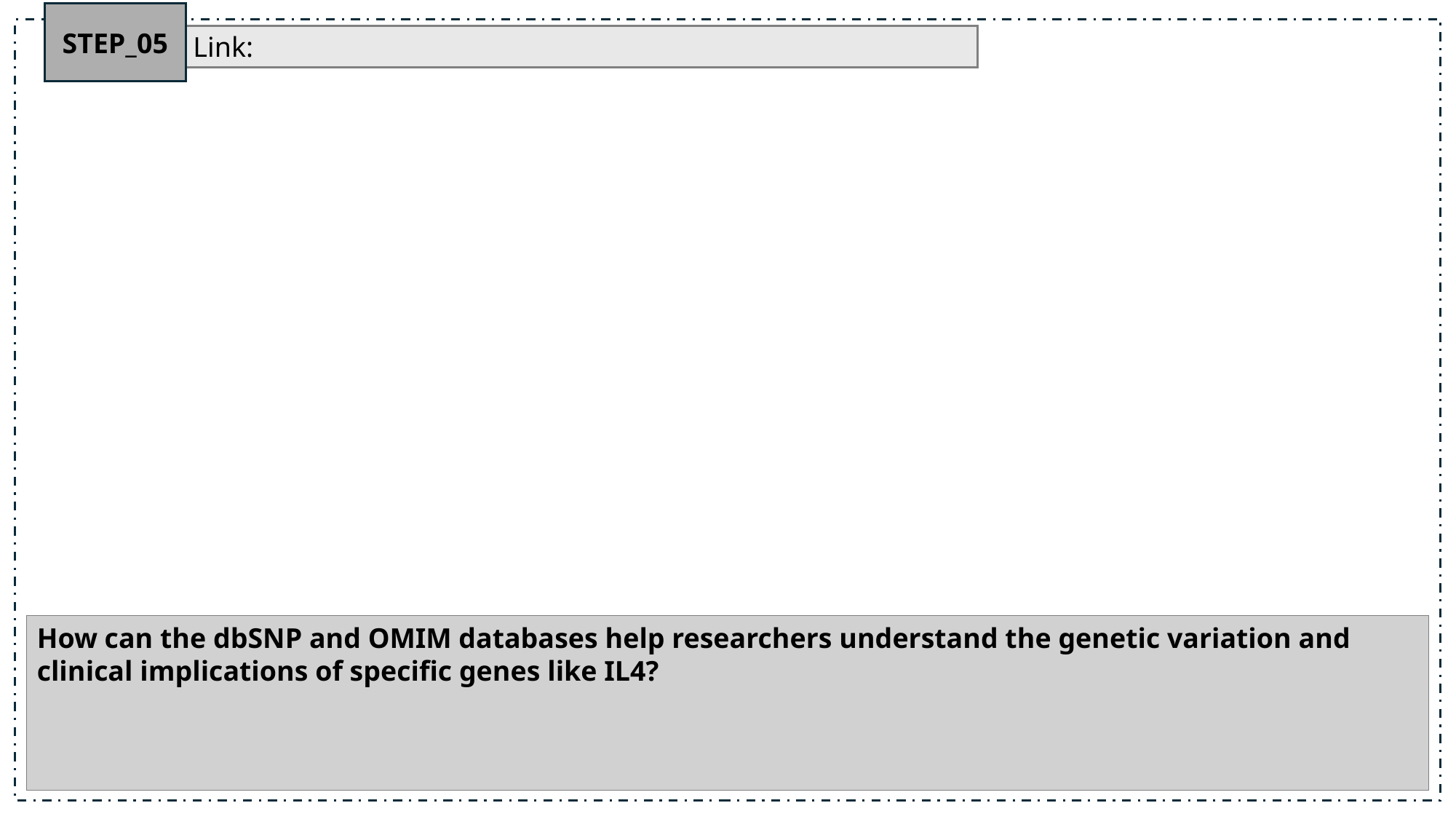

STEP_05
Link:
How can the dbSNP and OMIM databases help researchers understand the genetic variation and clinical implications of specific genes like IL4?

## Slide 7
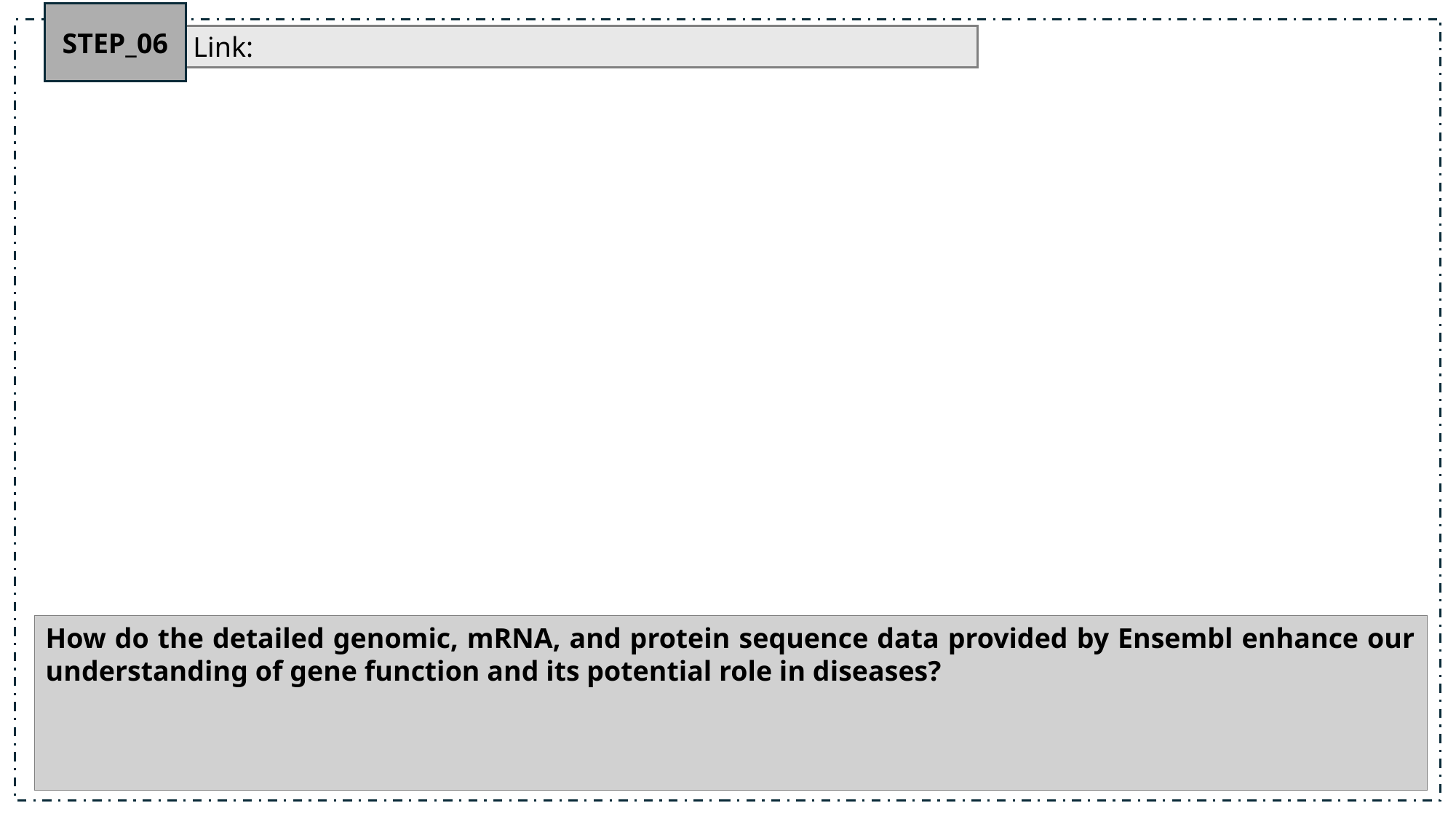

STEP_06
Link:
How do the detailed genomic, mRNA, and protein sequence data provided by Ensembl enhance our understanding of gene function and its potential role in diseases?

## Slide 8
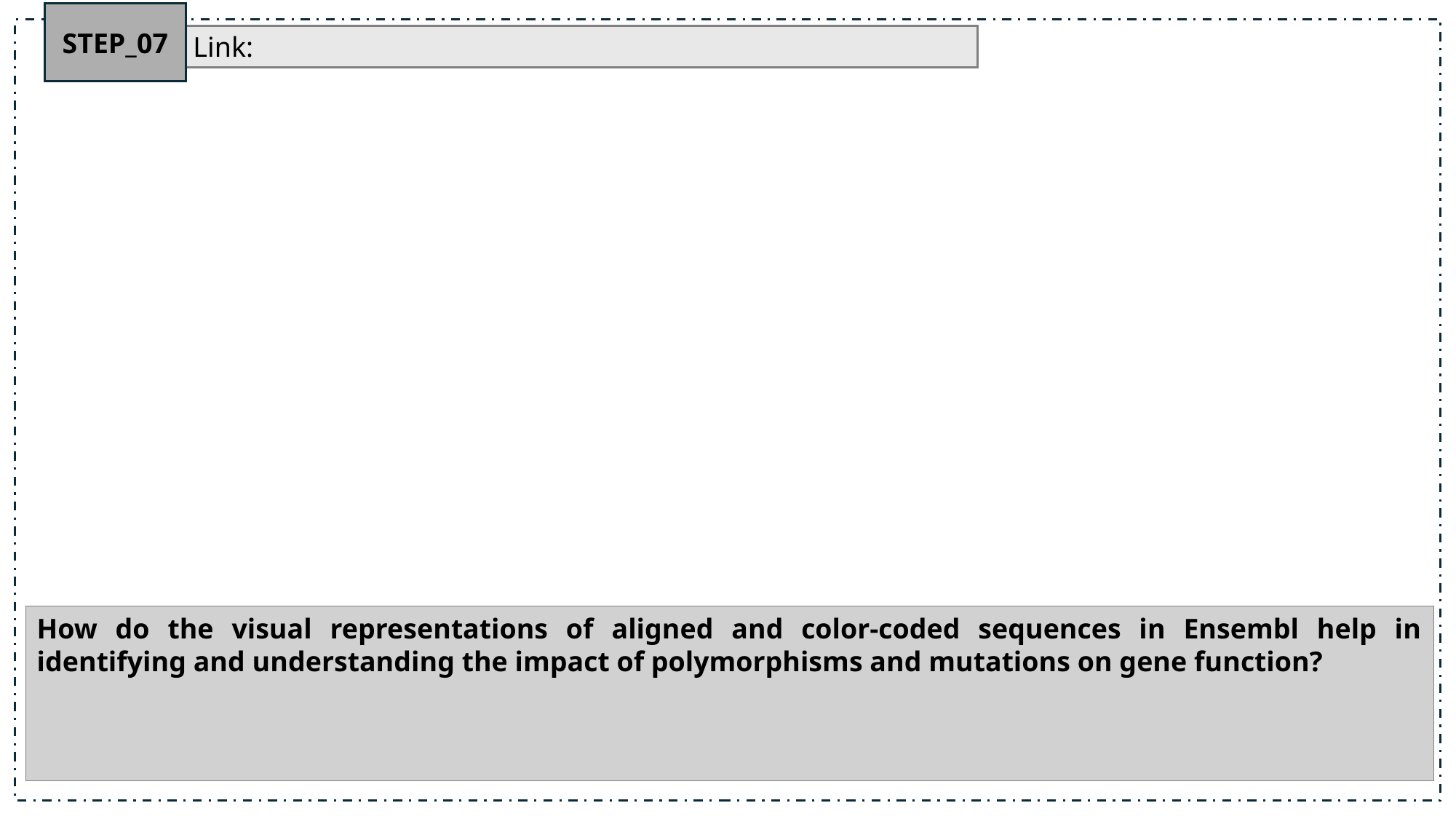

STEP_07
Link:
How do the visual representations of aligned and color-coded sequences in Ensembl help in identifying and understanding the impact of polymorphisms and mutations on gene function?

## Slide 9
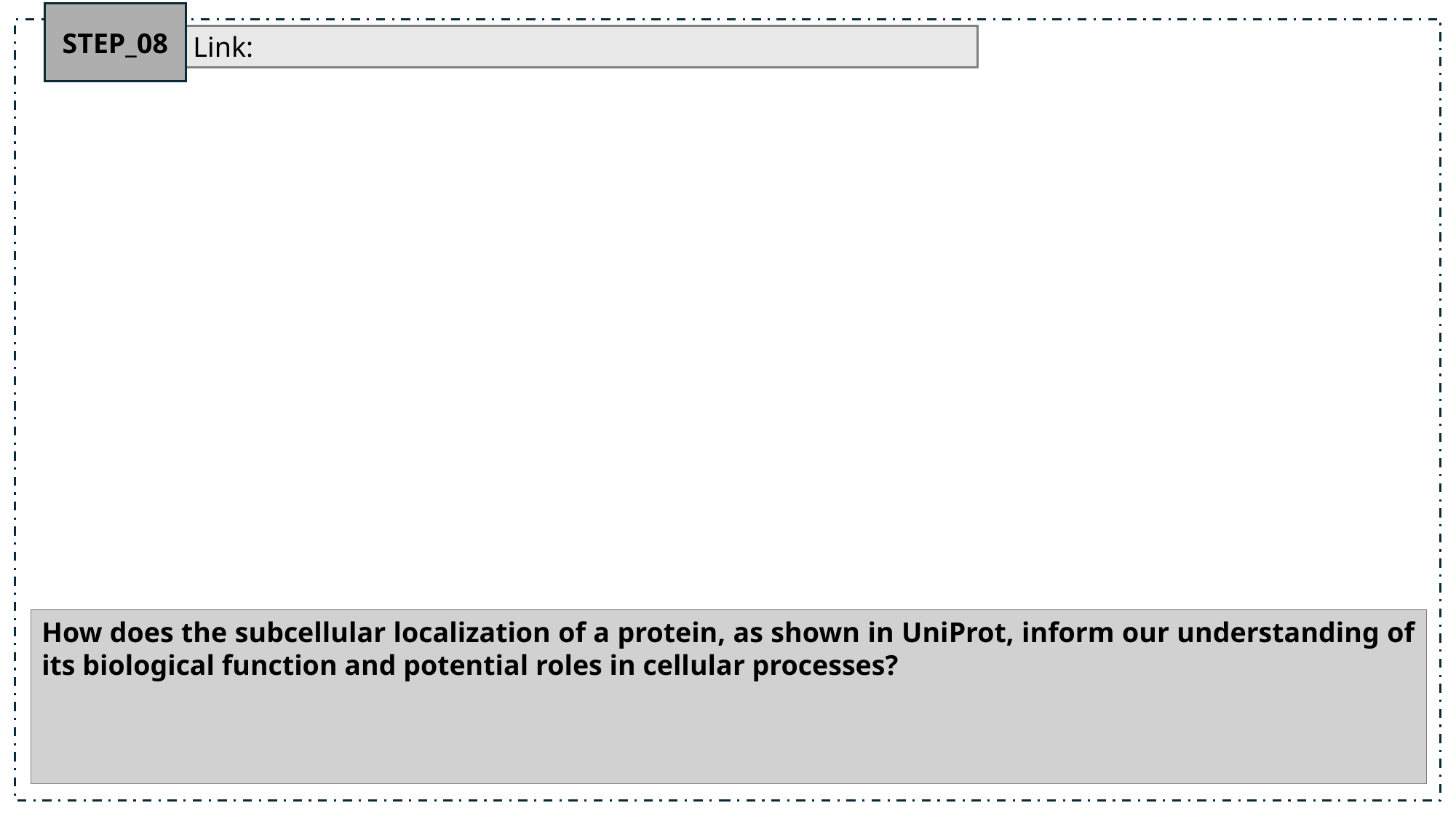

STEP_08
Link:
How does the subcellular localization of a protein, as shown in UniProt, inform our understanding of its biological function and potential roles in cellular processes?

## Slide 10
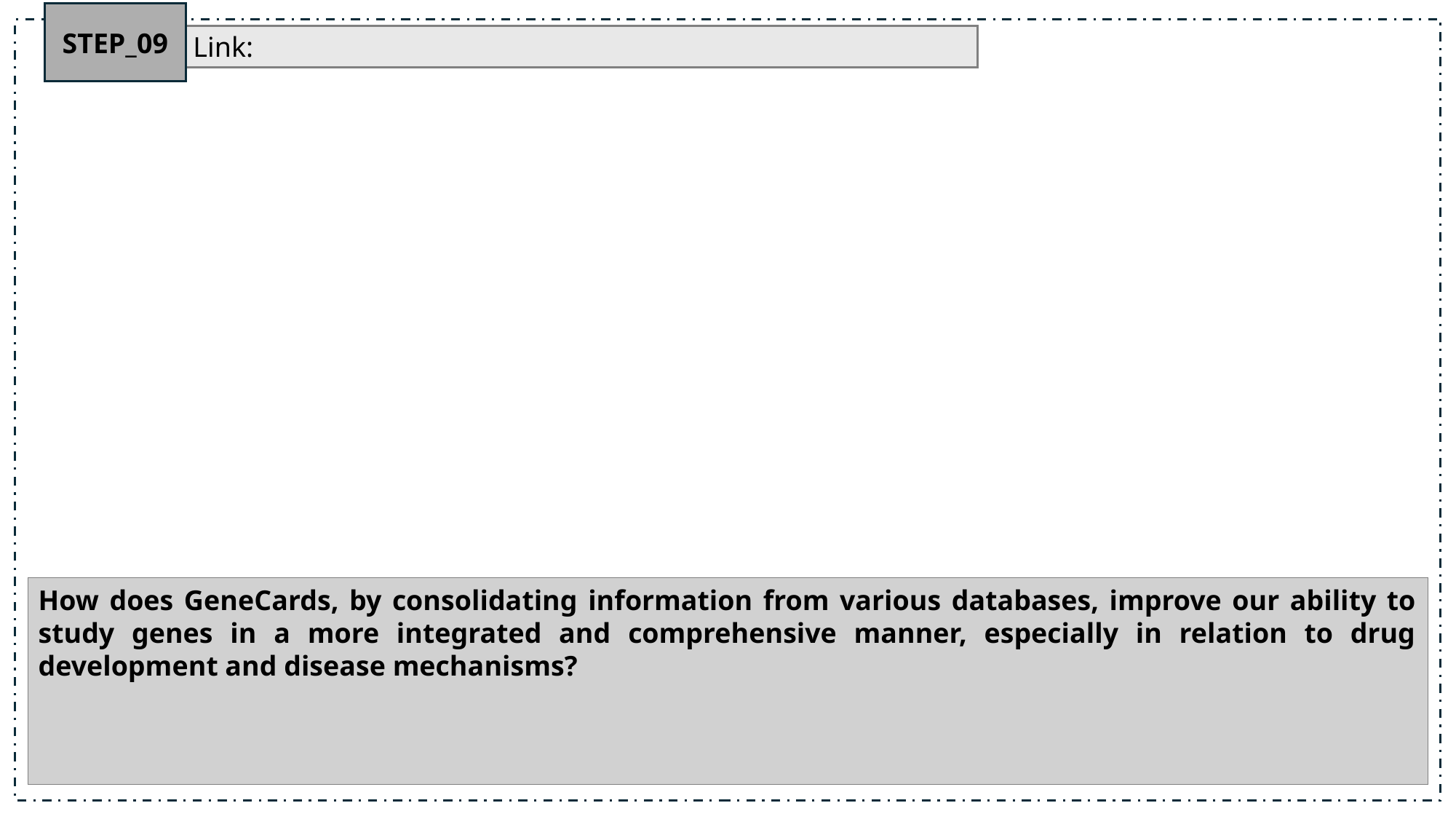

STEP_09
Link:
How does GeneCards, by consolidating information from various databases, improve our ability to study genes in a more integrated and comprehensive manner, especially in relation to drug development and disease mechanisms?

## Slide 11
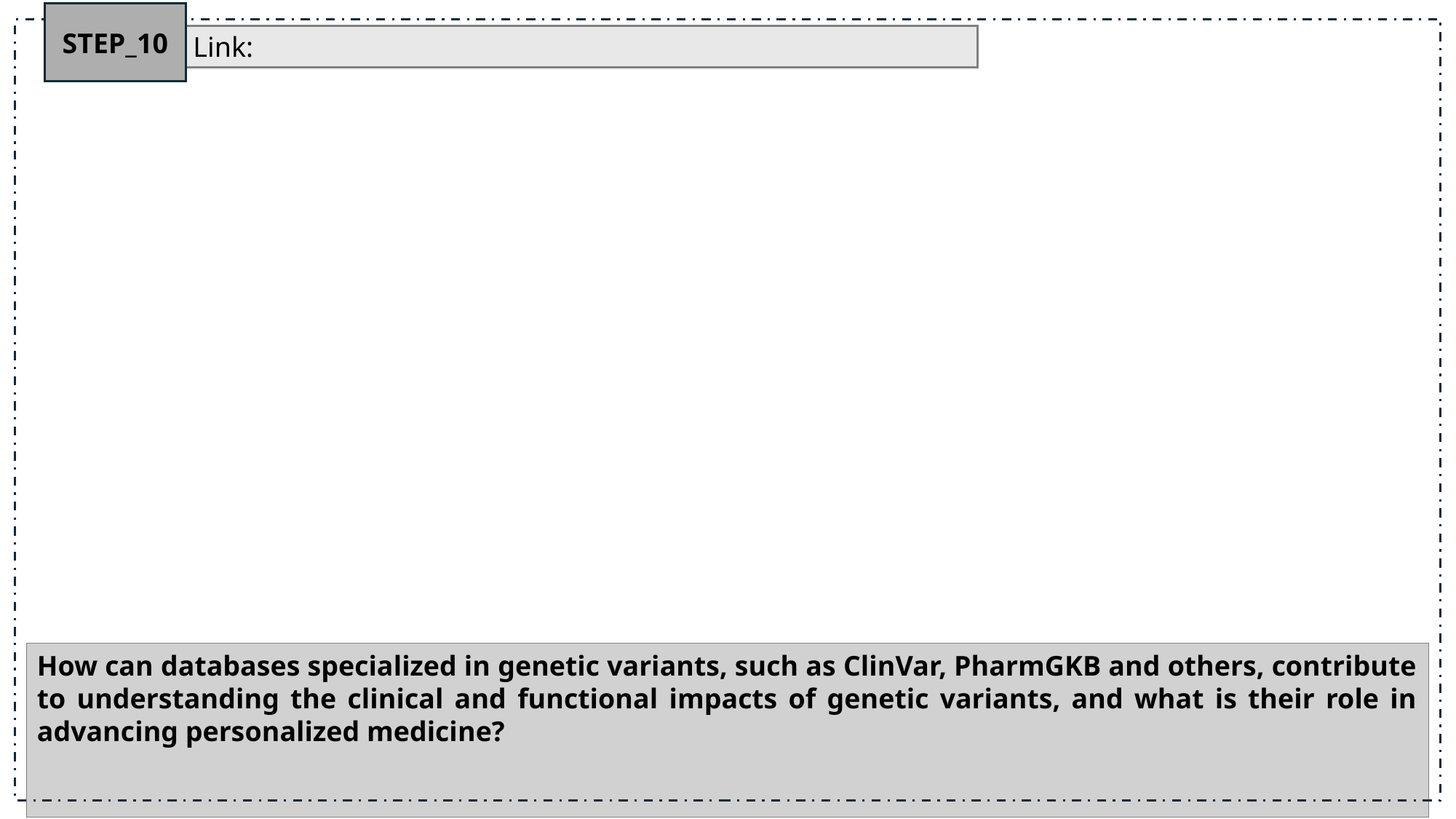

STEP_10
Link:
How can databases specialized in genetic variants, such as ClinVar, PharmGKB and others, contribute to understanding the clinical and functional impacts of genetic variants, and what is their role in advancing personalized medicine?

## Slide 12
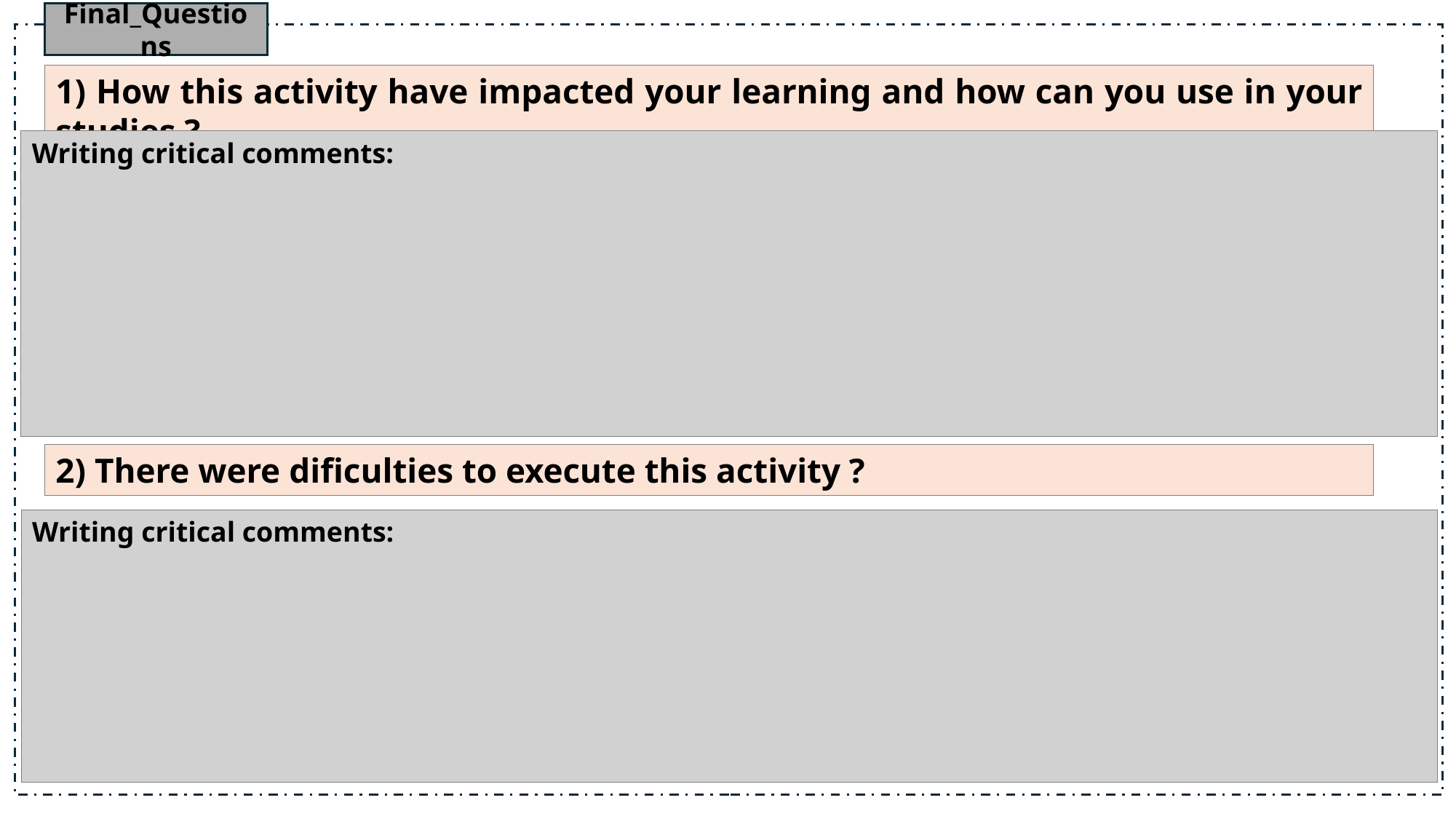

Final_Questions
1) How this activity have impacted your learning and how can you use in your studies ?
Writing critical comments:
2) There were dificulties to execute this activity ?
Writing critical comments:
